# NRF2‐REGγ‐ACADM/KLF15 Signaling Pathway Regulates the Browning of White Adipose Tissue to Modulate Obesity

**DOI:** 10.1002/advs.202509429

**Published:** 2025-09-19

**Authors:** Hui Chen, Qiujing Guan, Shuangming Gong, Yingying Du, Zhidan Zhang, Yan Liu, Lei Zhou, Lin Liu, Baoquan Xin, Yilan Guo, Hui Zhang, Ziyang Zhou, Tongchao Pei, Guohong Yu, Sokun Yin, Lei Li

**Affiliations:** ^1^ Shanghai Key Laboratory of Regulatory Biology Institute of Biomedical Sciences School of Life Sciences East China Normal University Shanghai 200241 China; ^2^ Department of Trauma‐Emergency & Critical Care Medicine Shanghai Fifth People's Hospital Fudan University Shanghai 200240 China; ^3^ Joint Center for Translational Medicine Shanghai Fifth People's Hospital Fudan University, and School of Life Science East China Normal University Shanghai 200241 China; ^4^ School of Life Sciences East China Normal University Shanghai 200241 China; ^5^ Chongqing Key Laboratory of Precision Optics Chongqing Institute of East China Normal University Chongqing 401120 China; ^6^ Department of Orthopedics Shanghai General Hospital Shanghai Jiao Tong University School of Medicine Shanghai 200080 China; ^7^ Department of Orthopaedic Oncology Changzheng Hospital Navy Medical University Shanghai 200003 China; ^8^ The Key Laboratory of Adolescent Health Assessment and Exercise Intervention of the Ministry of Education East China Normal University Shanghai 200241 China; ^9^ Department of Clinical Laboratory Shanghai Fifth People's Hospital Fudan University Shanghai 200240 China; ^10^ Department of Emergency Medicine Baoshan Second People's Hospital Baoshan College of Traditional Chinese Medicine 678000 Yunnan China; ^11^ Department of Emergency Medicine Luoping County People's Hospital 655800 Yunnan China; ^12^ Department of Preventive Treatment of disease by Traditional Chinese Medicine Putuo Hospital Affiliated to Shanghai University of Traditional Chinese Medicine Shanghai 200062 China

**Keywords:** PSME3, obesity, browning of white adipose tissue, ubiquitin‐independent protein degradation

## Abstract

Obesity is a significant risk factor for diabetes, cardiovascular diseases, and certain cancers, and manifests as excessive fat accumulation. The browning of white adipose tissue (WAT) represents one of the most promising strategies for preventing and treating obesity and metabolic diseases. To date, an increasing number of studies have focused on key molecular mechanisms regulating fat thermogenesis, laying the foundation for effective intervention strategies. Here, REGγ expression is shown to be significantly upregulated in adipose tissue of obese individuals and in inguinal WAT (iWAT) of obese mice. Deficiency in REGγ expression reduces fat deposition, increases energy expenditure in adipose tissue, and protects mice from HFD‐induced obesity and insulin resistance. Mechanistically, REGγ expression regulates browning of WAT by modulating ACADM and KLF15‐UCP1 signaling in a ubiquitin‐independent degradation manner. Overactivation of the NRF2‐REGγ axis facilitates adipose tissue function to cause obesity. Notably, inhibition of REGγ in the iWAT alleviates HFD‐induced obesity, thereby identifying REGγ as a latent target for obesity treatment. Together, the findings provide new targets for intervening in obesity and might ultimately offer new options for treating obesity.

## Graphical abstract

This study identifies that REGγ is elevated in obese adipose tissue. REGγ modulates WAT browning via ubiquitin‐independent ACADM/KLF15–UCP1 degradation. Overactivation of the NRF2–REGγ axis facilitates adipose tissue function to cause obesity. Inhibition of REGγ in the iWAT alleviates HFD‐induced obesity. These findings provide new targets for intervening in obesity and might ultimately offer new options for treating obesity.

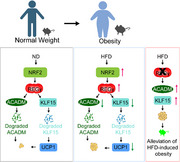

## 1 Introduction

Obesity is a common disease and a significant risk factor for diabetes, cardiovascular diseases, and certain cancers, and its prevalence has been increasing.^[^ ^1^ ^]^ Obesity manifests as excessive fat accumulation.^[^ ^2^ ^]^ When energy intake exceeds the energy needed to maintain life and daily activities, excessive energy is stored as fat, primarily in adipose tissue. Hence, reducing obesity requires interventions related to both energy intake and expenditure.

Beige adipose tissue is an intermediate type of adipose tissue found within white adipose tissue (WAT). The increased expression of UCP1 in WAT leads to energy expenditure and reduces lipid accumulation within adipocytes, resembling classic brown adipocyte functions. This phenomenon of brown‐like adipocytes appearing within WAT is termed “ browning of white adipose tissue”.^[^ ^3^, ^4^ ^]^ Beige adipocytes express key thermogenic genes (*Ucp1*, *Cidea*, *Pgc‐1α*).^[^ ^5^, ^6^ ^]^ The expression of UCP1 in WAT leads to energy expenditure and reduces lipid accumulation within adipocytes, resembling classic brown adipocyte functions. Notably, Cd137 and Tmem26 have been identified as surface markers for beige adipocyte precursors.^[^ ^5^ ^]^ The unique thermogenic capacity of brown and beige fat is due to a high density of mitochondria and the expression of uncoupling protein 1 (UCP1) in these thermogenic adipocytes.^[^ ^7^, ^8^ ^]^ Animal studies have shown that browning of white adipose tissue promotes weight loss, improves insulin resistance, and corrects hyperlipidemia associated with obesity, suggesting its potential in managing obesity and its complications.^[^ ^9^ ^]^ Thus, browning of white adipose tissue represents one of the most promising strategies for preventing and treating obesity and metabolic diseases.

Proteasome activator complex subunit 3 (PSME3), also known as REGγ, originally identified in the serum of systemic lupus erythematosus patients and named Ki antigen,^[^ ^10^ ^]^ binds to the 20S proteasome to form the REGγ‐proteasome complex and exerts important biological functions in many physiological and pathological processes.^[^ ^11^, ^12^, ^13^, ^14^, ^15^, ^16^, ^17^, ^18^, ^19^, ^20^, ^21^, ^22^, ^23^ ^]^ Our previous studies revealed that *REGγ* expression deficiency protects against high‐fat‐diet‐induced hepatic steatosis in mice.^[^ ^23^ ^]^ Interestingly, we also reported that the REGγ knockout mouse developed smaller body sizes.^[^ ^11^ ^]^ Thus, we speculate that REGγ may be involved in the regulation of adipose tissue deposition. However, further investigations are needed to fully elucidate the biological functions of REGγ in the browning of WAT to modulate obesity.

In this study, we discovered that REGγ is an important regulatory factor for the browning of WAT, thereby affecting overall metabolic balance. These results were supported by clinical samples, animal models, and at the molecular and cellular levels. We expect that this information will provide new targets for intervening in obesity and might ultimately offer new options for treating obesity.

## 2 Results

### 2.1 REGγ Plays an Important Role in Promoting Obesity

**Figure 1:**
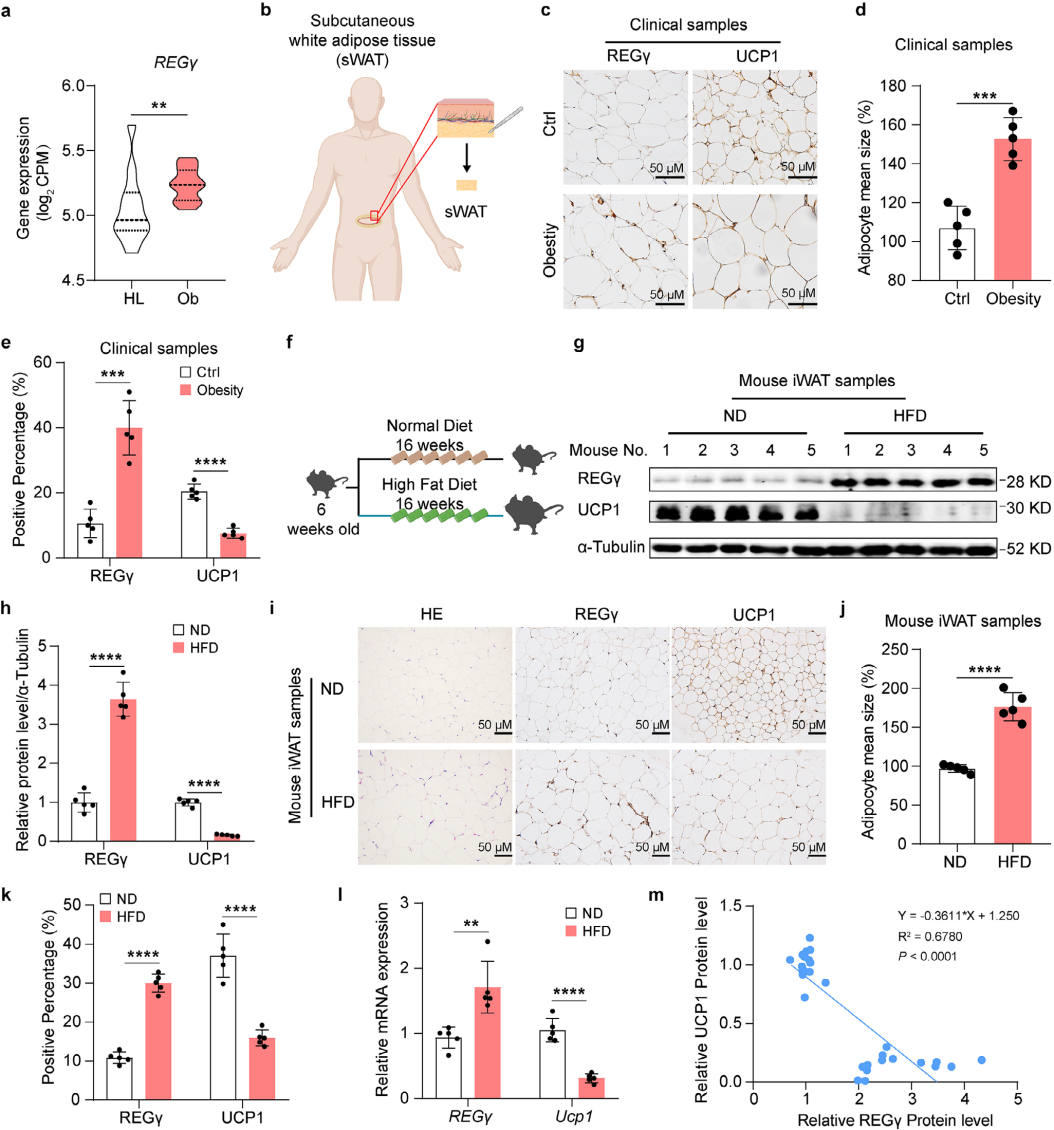
REGγ expression is significantly upregulated in adipose tissue of obese individuals and in the iWAT of obese mice. a) Gene expression of *REGγ* in human abdominal sWAT. Data were extracted from GSE159924. HL, healthy lean normal, *n* = 12; Ob, obese, *n*  =  21. b) Schematic diagram of sampling locations for obese and control adipose tissues. c‐e) Immunohistochemical (IHC) analysis of REGγ and UCP1 expression in control (Ctrl) and obese (Ob) individuals (c). Scale bar, 50 µm. Statistical results of adipocyte size (d) and quantification of REGγ and UCP1 protein expression (*n* = 5) (e). f) Schematic diagram of the construction of an obesity model induced by an HFD in mice. g‐h) Western blot analysis of REGγ, UCP1, and α‐Tubulin expression in inguinal white adipose tissue (iWAT) from the mice fed a normal diet (ND) or a high‐fat diet (HFD) for 12 weeks (g). Quantification of REGγ and UCP1 protein expression (*n* = 5) (h). i–k) Immunohistochemical analysis of REGγ and UCP1 expression in iWAT from mice fed an ND or an HFD for 12 weeks (i). Statistical results of adipocyte size (j) and quantification of REGγ and UCP1 protein expression (*n* = 5) (k). Scale bar, 50 µm. l) qRT‐PCR analysis of *REGγ* and *Ucp1* expression in iWAT from mice fed an ND or an HFD for 12 weeks (*n* = 5). m) Correlation of the protein expression levels of REGγ and UCP1 in iWAT from the mice (*n* = 30). Statistical significance was assessed by unpaired Student's t test (a, d, e, h, j, k, and l). Values were presented as Pearson's r correlation coefficient (m). *p < 0.05, **p < 0.01, ***p < 0.001, ****p < 0.0001.

Obesity manifests as excessive fat accumulation.^[^ ^2^ ^]^ In our previous studies, we have revealed that REGγ knockout mice developed a smaller body size and that *REGγ* expression deficiency protects mice against high‐fat‐diet (HFD)‐induced hepatic steatosis.^[^ ^11^, ^23^ ^]^ Here, we compared obese individuals (*n* = 21) with healthy controls (*n* = 12) and found that gene expression of *REGγ* was elevated in subcutaneous white adipose tissue (sWAT) in obese individuals from the GEO database (GSE159924) (**Figure** **1**a), suggesting that REGγ expression may contribute to the obesity phenotype. We subsequently collected sWAT from obese individuals (*n* = 5, body mass index (BMI) ≥ 30) and the sWATs from normal weight individuals (*n* = 5, 18 ≤ BMI < 25) obtained from Shanghai Fifth People's Hospital (Figure 1b). Compared with those of the control individuals, the protein expression of REGγ was increased, and UCP1 was decreased in adipose tissue of obese individuals (Figure 1c–e). Furthermore, we observed similar results in mice (Figure 1f–k). Similarly, mRNA expression of *REGγ* was increased, and that of *Ucp1* was reduced in obese mice compared with control mice (Figure 1l). Consistently, we found that protein expression of REGγ was negatively correlated with UCP1 in mice (Figure 1m), indicating that REGγ might play an important role in promoting obesity.

### 2.2 REGγ Deficiency Reduces Fat Deposition and Increases Energy Expenditure in Mice

To investigate the effect of REGγ expression on obesity, we analyzed the phenotypes of 4 to 24‐week‐old C57BL/6 wild‐type mice (WT mice) and *REGγ* knockout mice (KO mice) fed a normal diet. Compared with WT mice, *REGγ* KO mice presented with a smaller body size, reduced body fat content, a slight decrease in lean mass, improved glucose tolerance, and increased insulin sensitivity at 24 weeks of age (Figure S1a–e, Supporting Information). Furthermore, metabolic cage experiments revealed increased O_2_ consumption and CO_2_ production in *REGγ* KO mice, along with enhanced cold tolerance (Figure S1f,g, Supporting Information). We further observed significantly decreased volumes and weights of interscapular brown adipose tissue (BAT), inguinal white adipose tissue (iWAT), and epididymal white adipose tissue (eWAT) in *REGγ* KO mice compared to the WT mice (Figure S1h–j, Supporting Information). Thus, whole‐body knockout of REGγ expression led to a marked reduction in fat deposition and an increase in energy expenditure in mice.

**Figure 2:**
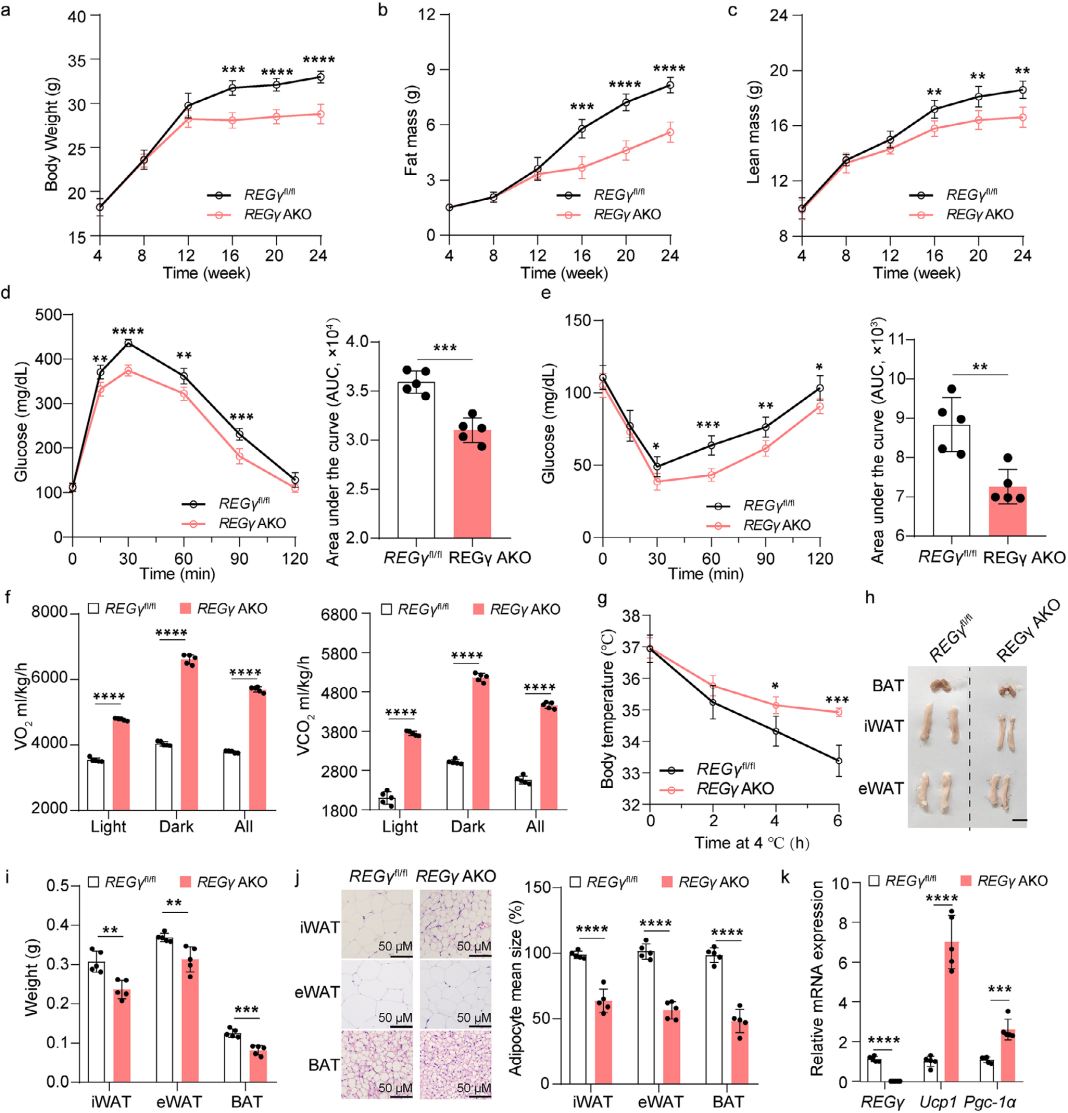
REGγ deficiency reduced fat deposition and increased energy expenditure in adipose tissue‐specific knockout mice. a‐c) Changes in body weight (a), fat mass (b), and lean mass (c) in 4‐week‐old to 24‐week‐old *REGγ* ^fl/fl^ and *REGγ* ^fl/fl^ *Adipoq*‐cre (REGγ AKO) mice (*n* = 5). d) Glucose tolerance test in 24‐week‐old *REGγ* ^fl/fl^ and *REGγ* AKO mice (*n* = 5). e) Insulin tolerance test in 24‐week‐old *REGγ* ^fl/fl^ and *REGγ* AKO mice (*n* = 5). f) Whole‐body oxygen consumption and carbon dioxide consumption analysis results of 24‐week‐old *REGγ* ^fl/fl^ and *REGγ* AKO mice (*n* = 5). g) Cold tolerance analysis of 24‐week‐old *REGγ* ^fl/fl^ and *REGγ* AKO mice (*n* = 5). h) Representative images of BAT, eWAT, and iWAT from 24‐week‐old *REGγ* ^fl/fl^ and *REGγ* AKO mice (*n* = 5). Scale bar, 1 cm. i) Weights of iWAT, eWAT, and BAT from 24‐week‐old *REGγ* ^fl/fl^ and *REGγ* AKO mice (*n* = 5). j) Representative haematoxylin and eosin (H&E) staining and quantification in iWAT, eWAT, and BAT from 24‐week‐old *REGγ* ^fl/fl^ and *REGγ* AKO mice (*n* = 5). Scale bar, 50 µm. k) qRT‐PCR analysis of expression of *REGγ*, and thermogenic genes (*Ucp1* and *Pgc‐1α*) in iWAT from 24‐week‐old *REGγ* ^fl/fl^ and *REGγ* AKO mice (*n* = 5). Statistical significance was assessed by two‐way ANOVA (a–e, and g), or unpaired Student's t test (f, i, j, and k). *p < 0.05, **p < 0.01, ***p < 0.001, ****p<0.0001. *REGγ* ^fl/fl^ *Adipoq*‐cre, AKO.

To investigate whether this phenotype is specific to adipose tissue, we utilized the Cre‐LoxP system to generate *REGγ* adipose tissue‐specific knockout (*REGγ* ^fl/fl^ *Adipoq*‐cre, AKO) mice. At 24 weeks of age, *REGγ* AKO mice had a smaller body size, reduced body fat content, improved glucose tolerance, and increased insulin sensitivity than *REGγ* ^fl/fl^ mice (**Figure** **2**a–e). The *REGγ* AKO mice also presented increased O_2_ consumption, CO_2_ production, and cold tolerance compared to *REGγ* ^fl/fl^ mice (Figure 2f,g). Moreover, we observed a significant reduction in the volumes and weights of iWAT, eWAT, and BAT in *REGγ* AKO mice compared to *REGγ* ^fl/fl^ mice (Figure 2h–j). These findings indicate that adipose tissue‐specific knockout of *REGγ* resulted in reduced fat deposition and enhanced energy expenditure in mice, suggesting a role for REGγ expression in regulating browning of WAT.

To further explore the role of REGγ in regulating browning of WAT, we examined mRNA expression levels of *REGγ* and thermogenic genes (*Ucp1* and *Pgc‐1α*) in the iWAT of *REGγ* KO and AKO mice, as well as their respective controls. Transcription of thermogenic genes was all increased (Figure 2k; Figure S1k, Supporting Information). Therefore, REGγ participates in the regulation of WAT browning.

### 2.3 REGγ Expression Promotes High‐Fat Diet (HFD)‐Induced Obesity and Insulin Resistance

**Figure 3:**
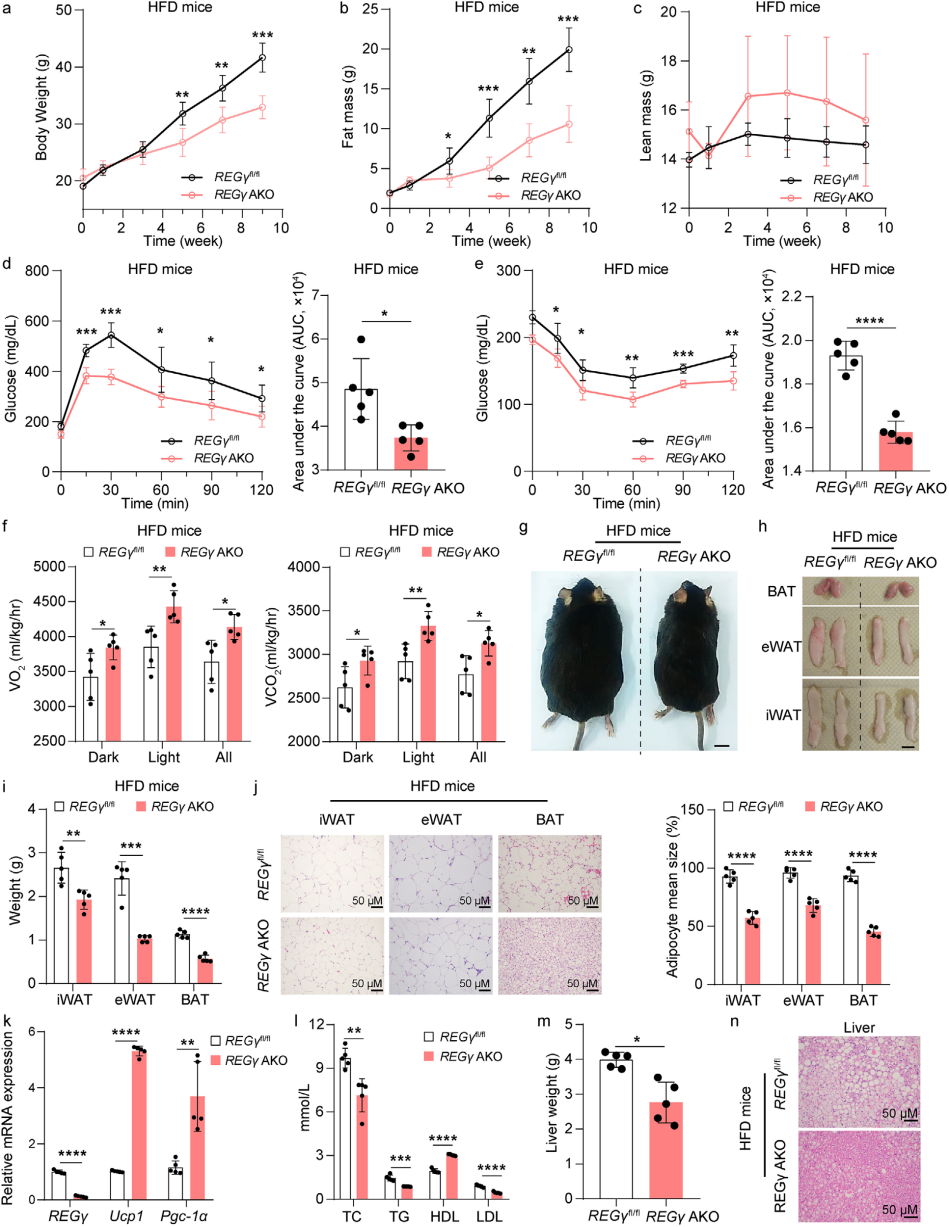
Deficiency of REGγ expression protects mice from HFD‐induced obesity and insulin resistance. a‐c) Changes in body weight (a), fat mass (b), and lean mass (c) in *REGγ* ^fl/fl^ and *REGγ* AKO mice fed a HFD (60%, ResearchDiet, D12492) for 12 weeks (*n* = 5). d) Glucose tolerance test in *REGγ* ^fl/fl^ and *REGγ* AKO mice fed a HFD for 12 weeks (*n* = 5). e) Insulin tolerance test in *REGγ* ^fl/fl^ and *REGγ* AKO mice fed a HFD for 12 weeks (*n* = 5). f) Whole‐body oxygen consumption and carbon dioxide consumption analysis results of *REGγ* ^fl/fl^ and *REGγ* AKO mice fed HFD for 4 weeks (*n* = 5). g) Representative images of *REGγ* ^fl/fl^ and *REGγ* AKO mice fed a HFD for 12 weeks (*n* = 5). h) Representative images of BAT, eWAT, and iWAT from *REGγ* ^fl/fl^ and *REGγ* AKO mice fed a HFD for 12 weeks (*n* = 5). Scale bar, 1 cm. i) Weights of iWAT, eWAT, and BAT from *REGγ* ^fl/fl^ and *REGγ* AKO mice fed a HFD for 12 weeks (*n* = 5). j) Representative H&E staining and quantification of iWAT, eWAT, and BAT from *REGγ* ^fl/fl^ and *REGγ* AKO mice fed a HFD for 12 weeks (*n* = 5). Scale bar, 50 µm. k) qRT‐PCR analysis of expression of *REGγ* and thermogenic genes (*Ucp1* and *Pgc‐1α*) in iWAT from *REGγ* ^fl/fl^ and *REGγ* AKO mice fed a HFD for 12 weeks (*n* = 5). l) ELISA analysis of blood lipids, including total cholesterol (TC), triglycerides (TG), high‐density lipoprotein (HDL), and low‐density lipoprotein (LDL), in the serum from *REGγ* ^fl/fl^ and *REGγ* AKO mice fed a HFD for 12 weeks (*n* = 5). m) Weights of livers in *REGγ* ^fl/fl^ and *REGγ* AKO mice fed a HFD for 12 weeks (*n* = 5). n) Representative H&E staining in livers from *REGγ* ^fl/fl^ and *REGγ* AKO mice fed a HFD for 12 weeks (*n* = 5). Statistical significance was assessed by two‐way ANOVA (a‐e), or unpaired Student's t test (f, i‐l, and m). *p < 0.05, **p < 0.01, ***p < 0.001, ****p < 0.0001. *REGγ* ^fl/fl^ *Adipoq*‐cre, AKO.

HFD‐induced obesity is mainly caused by adipose tissue dysfunctions. To explore the function of REGγ expression in HFD‐induced obesity, we fed mice an HFD at 6 weeks of age for 12 weeks. Compared with their respective controls, after feeding on an HFD for 12 weeks, *REGγ* KO and *REGγ* AKO mice presented with a smaller body size, reduced body fat content, improved glucose tolerance, and increased insulin sensitivity (**Figure** **3**a–e; Figure S2a–e, Supporting Information). However, the lean mass in *REGγ* KO and *REGγ* AKO mice exhibited no difference compared with their respective controls. Furthermore, metabolic cage experiments revealed increased O_2_ consumption and CO_2_ production in *REGγ* KO and *REGγ* AKO mice compared to their respective controls after 4 weeks on an HFD (Figure 3f; Figure S2f, Supporting Information). We subsequently observed significantly lower volumes and weights of BAT, iWAT, and eWAT in *REGγ* KO and *REGγ* AKO mice than in their respective controls after 12 weeks on an HFD (Figure 3g–j; Figure S2g–j, Supporting Information).

Furthermore, the mRNA expression levels of thermogenic genes (*Ucp1* and *Pgc‐1α*) were increased in iWAT tissue of *REGγ* KO and *REGγ* AKO mice on an HFD for 12 weeks, compared to their respective controls (Figure 3k; Figure S2k, Supporting Information). Meanwhile, triglyceride (TG), total cholesterol (TC), low‐density lipoprotein‐cholesterol (LDL) levels were decreased, and the high‐density lipoprotein (HDL) levels were increased in the serum of *REGγ* AKO mice on a HFD for 12 weeks (Figure 3l; Figure S2i, Supporting Information), accompanied by decreased liver weights and lipid infiltration, compared with controls (Figure 3m,n; Figure S2m,n, Supporting Information).

Otherwise, we have crossed LoxP‐Stop‐LoxP (LSL)‐*REGγ* ^fl/fl^ (Ctrl)^[^ ^20^, ^24^ ^]^ mice with *Adipoq*‐Cre mice to obtain *REGγ* AOE mice with adipose tissue‐specific overexpression of REGγ. We discovered that, compared with Ctrl mice, the *REGγ* AOE mice exhibited more severe obesity‐related phenotypes, including increased body weight, increased fat mass, increased glucose tolerance, increased insulin tolerance, decreased oxygen consumption, increased carbon dioxide production, increased fat and liver weights, and increased fat volume (Figure S3a–k, Supporting Information). Furthermore, we analyzed the protein expression of REGγ, ACADM, and UCP1 in iWAT from Ctrl and *REGγ* AOE mice fed a HFD for 13 weeks. We found that the protein and mRNA expression levels of Acadm and UCP1 were decreased in iWAT from Ctrl and *REGγ* AOE mice (Figure S3l,m, Supporting Information).

Overall, we conclude that REGγ expression deficiency protects mice from HFD‐induced obesity and insulin resistance.

### 2.4 REGγ Regulates ACADM Expression by Ubiquitin‐Independent Degradation to Induce Obesity

**Figure 4:**
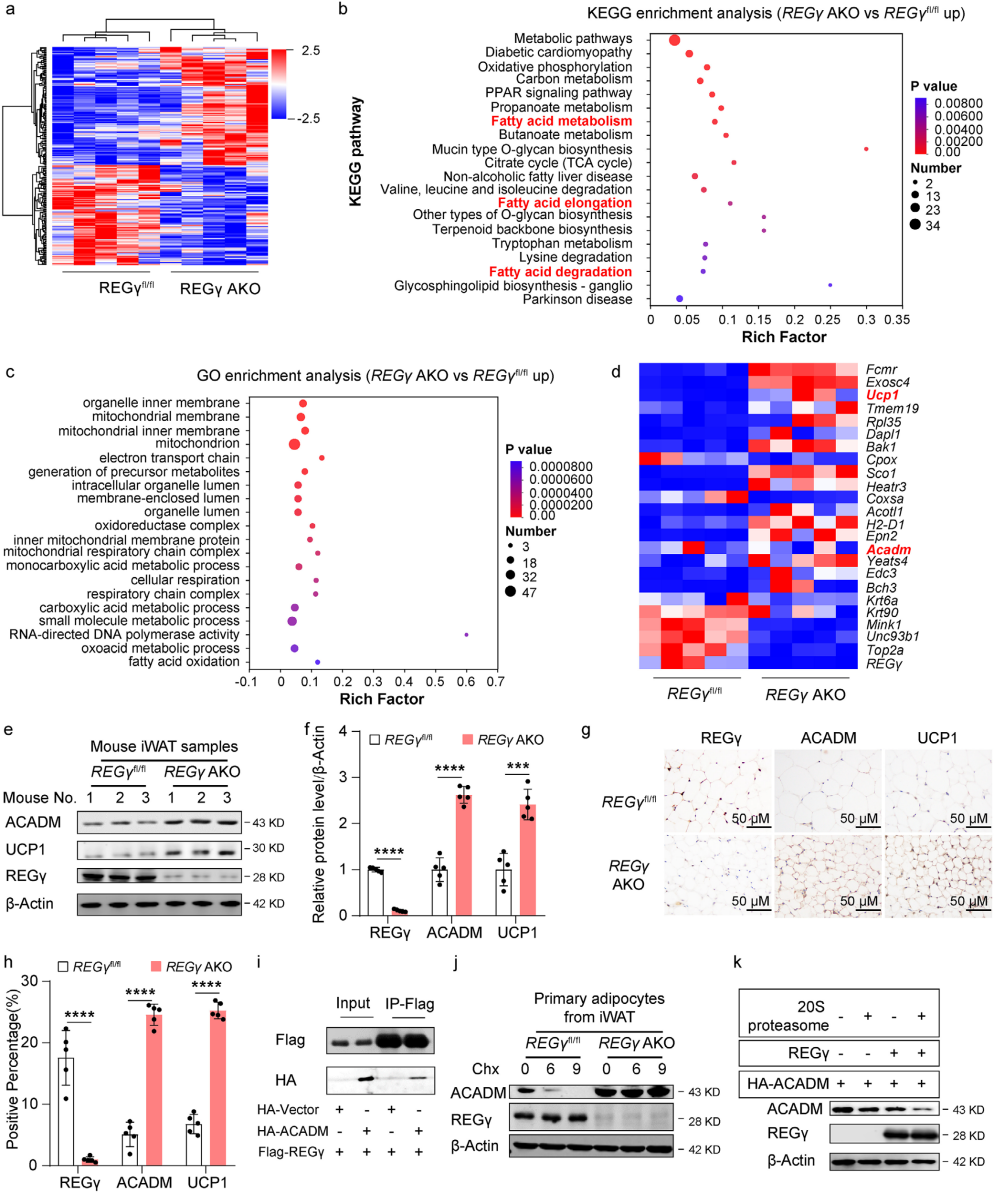
REGγ regulates ACADM expression via ubiquitin‐independent degradation to induce obesity. a) Heatmap of differentially expressed proteins in iWAT from 24‐week‐old *REGγ* ^fl/fl^ and *REGγ* AKO mice (*n* = 5). b‐c) KEGG (b) and GO (c) analyses of upregulated proteins in iWAT from 24‐week‐old *REGγ* ^fl/fl^ and *REGγ* AKO mice (*n* = 5). d) qRT‐PCR analysis of the top 20 upregulated and top 4 downregulated candidates (*n* = 5). e‐f) Western blot analysis of REGγ, UCP1, ACADM, and β‐Actin expression in iWAT from 24‐week‐old *REGγ* ^fl/fl^ and *REGγ* AKO mice (e). Quantification of REGγ, UCP1, and ACADM protein expression (f) (*n* = 5). g‐h) IHC analysis of REGγ, UCP1, and ACADM in iWAT from 24‐week‐old *REGγ* ^fl/fl^ and *REGγ* AKO mice (g). Scale bar, 50 µm. Quantification of REGγ, UCP1, and ACADM protein expression (h) (*n* = 5). i) Co‐immunoprecipitation (CO‐IP) analysis of REGγ and ACADM. j) Western blot analysis of ACADM, REGγ, and β‐Actin expression in primary adipocytes (obtained after six days of differentiation from adipocyte precursor cells of iWAT) from 4‐week‐old *REGγ* ^fl/fl^ and *REGγ* AKO mice after 0, 6, or 9 h of Cycloheximide (Chx) treatment (j). k) Western blot analysis of REGγ, ACADM, and β‐Actin after the degradation of ACADM in vitro. Statistical significance was assessed by an unpaired Student's t‐test (f and h). *p < 0.05, **p < 0.01, ***p < 0.001, ****p < 0.0001. *REGγ* ^fl/fl^ *Adipoq*‐cre, AKO.

To elucidate the mechanism by which REGγ regulates browning of WAT, we performed proteomic analysis in *REGγ* ^fl/fl^ and *REGγ* AKO mice. We observed significant changes in protein expression in metabolic pathways, particularly those related to mitochondria (**Figure** **4**a–c). We applied qRT‐PCR to analyze the gene expression of the top 15 upregulated and the top 6 genes downregulated proteins, and found no significant change in transcription of *Acadm*, a significantly increased protein in *REGγ* AKO mice (Figure 4d). ACADM, a lipid metabolism enzyme, catalyzes the first dehydrogenation step of β‐oxidation, playing a vital role in maintaining the body's energy balance and metabolic health.^[^ ^25^ ^]^ Thus, we hypothesize that REGγ expression may regulate ACADM protein degradation.

Next, we analyzed ACADM protein expression in iWAT of *REGγ* KO and *REGγ* AKO mice and control mice, and found significantly increased protein levels of ACADM and UCP1, which were consistent with the proteomic results (Figure 4e,f; Figure S4a,b, Supporting Information). Furthermore, we determined the protein and mRNA levels of *REGγ*, *Acadm*, and *Ucp1* in primary adipocytes from the iWAT of *REGγ* KO, *REGγ* AKO mice, and control mice and observed similar results as in mouse tissues (Figure S4c–e, Supporting Information). Moreover, the protein expression levels of ACADM and UCP1 were increased in iWAT from *REGγ* KO and *REGγ* AKO mice as determined by immunohistochemistry (IHC) (Figure 4g,h; Figure S4f,g, Supporting Information), which was consistent with the WB results.

Co‐immunoprecipitation(co‐IP) experiments demonstrated an interaction between REGγ and ACADM (Figure 4i). Additionally, we compared the degradation of ACADM in primary adipocytes between control and *REGγ* KO mice after Cycloheximide (Chx) treatment, revealing an increased ACADM stability in cells after the deletion of REGγ (Figure 4j; Figure S4h–j, Supporting Information). In vitro protein degradation experiment revealed that REGγ can directly degrade ACADM in a ubiquitin‐independent degradation (Figure 4k), suggesting that REGγ regulates ACADM via a ubiquitin‐independent degradation pathway (Figure S4k, Supporting Information). WB analysis revealed that the protein expression of ACADM and UCP1 was greater in iWAT in *REGγ* KO and *REGγ* AKO mice than in control mice, after feeding on an HFD for 12 weeks (Figure S4l,m, Supporting Information).

Next, we knocked down *Acadm* using two different sequences in primary adipocytes from iWAT of WT and *REGγ* ^fl/fl^ mice and found that sequence sh*Acadm*‐1# was more effective (Figure S5a,b, Supporting Information). Subsequently, we purchased the AAV virus carrying sh*Acadm*‐1# (hereinafter referred to as AAV sh*Acadm*) and shN (negative control), and used them for therapeutic experiments in *REGγ* AKO (*REGγ*‐specific knockout) animals (Figure S5c, Supporting Information). We discovered that, compared with *REGγ* AKO mice, the AAV sh*Acadm*‐treated *REGγ* AKO mice exhibited more severe obesity‐related phenotypes, including increased body weight, increased fat mass, increased glucose tolerance, increased insulin tolerance, decreased oxygen consumption, increased carbon dioxide production, increased fat and liver weights, and increased fat volume (Figure S5d–l, Supporting Information). Furthermore, we analyzed the protein expression of REGγ, ACADM, and UCP1 in iWAT from *REGγ* AKO and the AAV sh*Acadm*‐treated *REGγ* AKO mice fed a HFD for 12 weeks. We found that the protein and mRNA expression levels of ACADM and UCP1 were decreased in iWAT from the AAV sh*Acadm*‐treated *REGγ* AKO mice compared to *REGγ* AKO mice (Figure S5m–o, Supporting Information).

Therefore, REGγ regulates browning of WAT by degrading ACADM through ubiquitin‐ and ATP‐independent protein degradation pathways to induce obesity.

### 2.5 REGγ Modulates KLF15 Expression via Ubiquitin‐Independent Degradation to Induce Obesity

The increased expression of UCP1 in WAT leads to energy expenditure and reduced lipid accumulation in adipocytes, resembling classic brown adipocyte functions.^[^ ^3^, ^4^ ^]^ In our study, we found that the protein and mRNA levels of *Ucp1* were increased in iWAT and from *REGγ* KO and *REGγ* AKO mice, as well as in primary adipocytes from iWAT of *REGγ* KO and *REGγ* AKO mice compared with controls (Figure 4d–h; Figure S4a–g, Supporting Information), indicating that REGγ might regulate the expression of UCP1 in browning of WAT to induce obesity. In our previous study, we reported that REGγ enhances the transcriptional activity of NF‐κB by directly binding to and destabilizing KLF2.^[^ ^26^ ^]^ Moreover, a literature review revealed that KLF15 is a positive transcriptional regulator of *Ucp1*.^[^ ^27^ ^]^ Thus, we determined the protein and mRNA levels of *Klf2*, *Klf4*, and *Klf15* in WT and *REGγ* KO primary adipocytes from the iWAT of WT and *REGγ* KO mice. We found that the protein level of KLF15 was increased in *REGγ* KO primary adipocytes, whereas there was no change in mRNA level (Figure S6a,b, Supporting Information), indicating that REGγ might regulate KLF15 at the protein level to suppress the expression of *Ucp1*.

**Figure 5:**
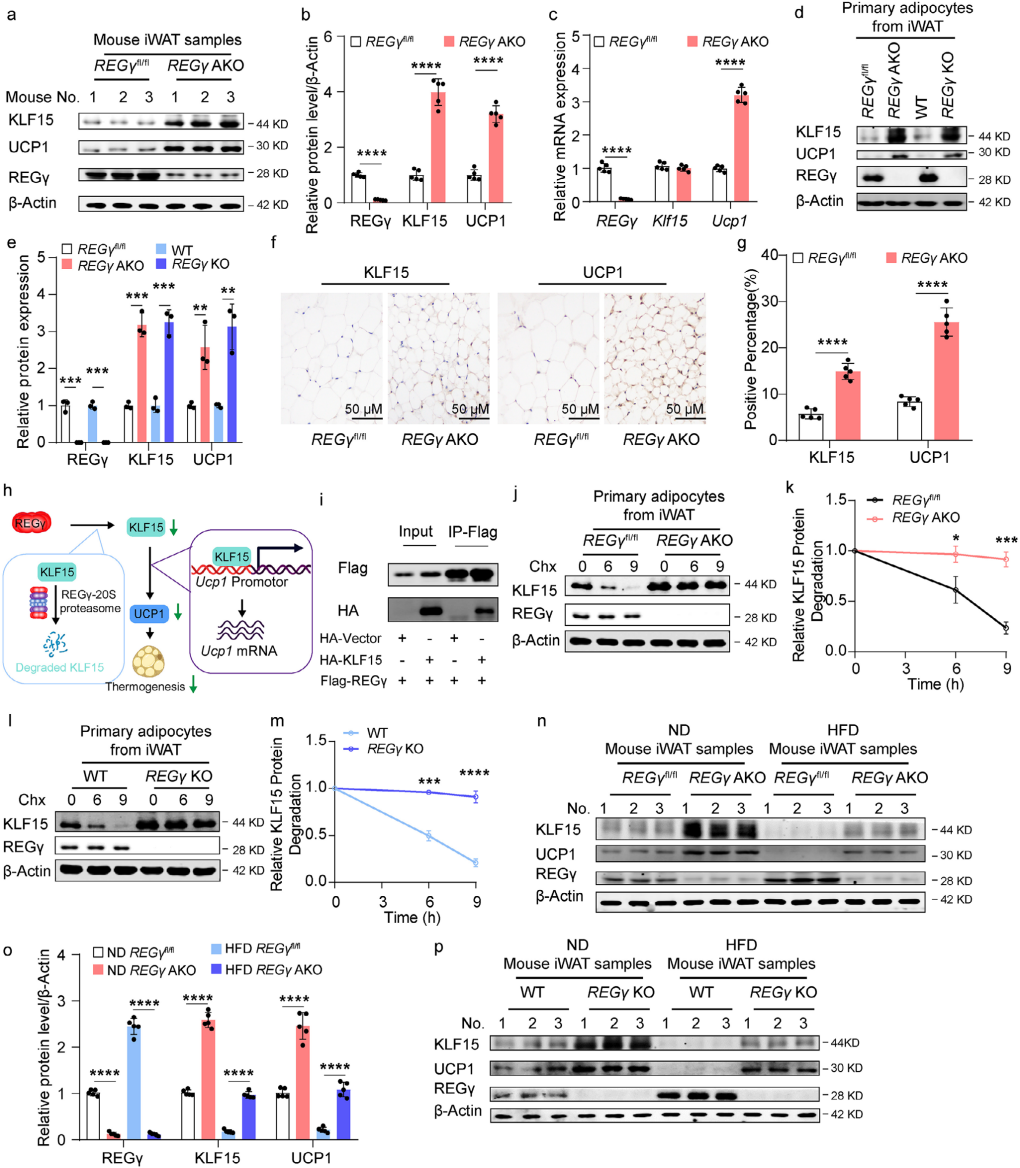
REGγ modulates KLF15 expression via ubiquitin‐independent degradation to induce obesity. a, b) Western blot analysis of REGγ, UCP1, KLF15, and β‐Actin expression in iWAT from 24‐week‐old *REGγ* ^fl/fl^ and *REGγ* AKO mice (a). Quantification of REGγ, UCP1, and ACADM protein expression (b) (*n* = 5). c) qRT‐PCR analysis of REGγ, KLF15 and UCP1 in iWAT from 24‐week‐old *REGγ* ^fl/fl^ and *REGγ* AKO mice (*n* = 5). d, e) Western blot analysis of REGγ, UCP1, KLF15, and β‐Actin in primary adipocytes from 4‐week‐old *REGγ* ^fl/fl^, *REGγ* AKO, WT, and *REGγ* KO mice (d). Quantification of REGγ, KLF15, and UCP1 protein expression in primary adipocytes from 4‐week‐old *REGγ* ^fl/fl^, *REGγ* AKO, WT, and *REGγ* KO mice (e). f, g) IHC analysis of UCP1 and KLF15 expression in iWAT from 24‐week‐old *REGγ* ^fl/fl^ and *REGγ* AKO mice (f). Scale bar, 50 µm. Quantification of UCP1 and KLF15 protein expression (g) (*n* = 5). h) Hypothetical schematic diagram illustrating the involvement of REGγ in the regulation of thermogenesis through the degradation of KLF15 in a ubiquitin‐independent manner. i) Co‐immunoprecipitation (CO‐IP) analysis of REGγ and KLF15. j‐k) Western blot analysis of KLF15, REGγ, and β‐Actin expression in primary adipocytes from 4‐week‐old *REGγ* ^fl/fl^ and *REGγ* AKO mice after 0, 6, or 9 h of Cycloheximide (Chx) treatment (j). Quantification of degradation (k). l, m) Western blot analysis of KLF15, REGγ, and β‐Actin expression in primary adipocytes from 4‐week‐old WT and *REGγ* KO mice after 0, 6, or 9 h of Cycloheximide (Chx) treatment (l). Quantification of degradation (m). n) Western blot analysis of KLF15, UCP1, REGγ, and β‐Actin expression in iWAT from ND‐ or HFD‐fed *REGγ* ^fl/fl^ and *REGγ* AKO mice for 12 weeks (*n* = 5). o) Quantification of the protein expression of KLF15, UCP1, REGγ, and β‐Actin in iWAT from ND‐ or HFD‐fed *REGγ* ^fl/fl^ and *REGγ* AKO mice for 12 weeks (*n* = 5). Related toFigure 5n. p) Western blot analysis of KLF15, UCP1, REGγ, and β‐Actin expression in iWAT from ND‐ or HFD‐fed WT and *REGγ* KO mice for 12 weeks (*n* = 5). Statistical significance was assessed by unpaired Student's t test (b, c, e, g, k, and m). *p < 0.05, **p < 0.01, ***p < 0.001, ****p < 0.0001. *REGγ* ^fl/fl^ *Adipoq*‐cre, AKO.

Next, we analyzed the protein and mRNA levels of *Klf15* in the iWAT of *REGγ* KO and *REGγ* AKO mice and found that KLF15 was significantly increased at the protein level but not the mRNA level compared to control (**Figure** **5**a–c; Figure S6c–e, Supporting Information). Furthermore, the protein and mRNA expression of *REGγ*, *Klf15*, and *Ucp1* in primary adipocytes from the iWAT of *REGγ* KO and *REGγ* AKO mice showed similar patterns compared to control (Figure 4d,e). The protein expression of ACADM and UCP1 was increased in iWAT from *REGγ* KO and *REGγ* AKO mice as confirmed by IHC compared to control (Figure 5f,g; Figure S6f,g, Supporting Information), indicating REGγ regulates KLF15 at the protein level via ubiquitin‐independent degradation to reduce the transcription of *Ucp1* (Figure 5h).

Co‐IP experiments demonstrated an interaction between REGγ and KLF15 (Figure 5i). Comparing the degradation of KLF15 in control and *REGγ* KO primary adipocytes after Cycloheximide (Chx) treatment revealed increased KLF15 stability after deletion of REGγ (Figure 5j–m). In addition, the protein levels of ACADM and KLF15 were significantly increased in cells treated with MG132 (proteasome‐specific inhibitor) after knocking out REGγ (Figure S6h, Supporting Information). Expression of KLF15 and UCP1 proteins was increased in iWAT from *REGγ* KO and *REGγ* AKO mice after feeding HFD for 12 weeks, as shown by WB analysis (Figure 5n–p; Figure S6i, Supporting Information). Therefore, REGγ regulates browning of WAT by degrading KLF15 through ubiquitin‐ and ATP‐independent protein degradation pathways to induce obesity.

Furthermore, we analyzed the protein expression of REGγ, ACADM, KLF15, and UCP1 in iWAT from *REGγ* KO and *REGγ* AKO mice compared with their respective controls fed a HFD or ND for 12 weeks. We found that the protein expression levels of ACADM, KLF15, and UCP1 were higher in iWAT from *REGγ* KO and *REGγ* AKO mice than in those from control mice feeding on HFD for 12 weeks. Compared with those in ND‐fed mice, protein levels of ACADM, KLF15, and UCP1 were decreased, and the protein level of REGγ was increased in iWAT from *REGγ* ^fl/fl^ and WT mice feeding on HFD for 12 weeks (Figure S6j–m, Supporting Information).

Next, we knocked down *Klf15* using two different sequences in primary adipocytes from iWAT of WT and *REGγ* ^fl/fl^ mice and found that sequence sh*Klf15*‐1# was more effective (Figure S7a–b, Supporting Information). Subsequently, we purchased the AAV virus carrying sh*Klf15*‐1# (hereinafter referred to as AAV sh*Klf15*) and shN (negative control), and used them for therapeutic experiments in *REGγ* AKO (*REGγ*‐specific knockout) animals (Figure S7c, Supporting Information). We discovered that, compared with *REGγ* AKO mice, the AAV sh*Klf15*‐treated *REGγ* AKO mice exhibited more severe obesity‐related phenotypes, including increased body weight, increased fat mass, increased glucose tolerance, increased insulin tolerance, decreased oxygen consumption, increased carbon dioxide production, increased fat and liver weights, and increased fat volume (Figure S7d–l, Supporting Information). Furthermore, we analyzed the protein expression of REGγ, KLF15, and UCP1 in iWAT from REGγ AKO and the AAV sh*Klf15*‐treated *REGγ* AKO mice fed a HFD for 9 weeks. We found that the protein and mRNA expression levels of KLF15 and UCP1 were decreased in iWAT from the AAV sh*Klf15*‐treated *REGγ* AKO mice compared to *REGγ* AKO mice (Figure S7m–o, Supporting Information).

Overall, we revealed that REGγ regulates browning of WAT via ACADM and KLF15‐UCP1 signaling to modulate obesity.

### 2.6 NRF2 Regulates the Transcription of REGγ to Promote Obesity

**Figure 6:**
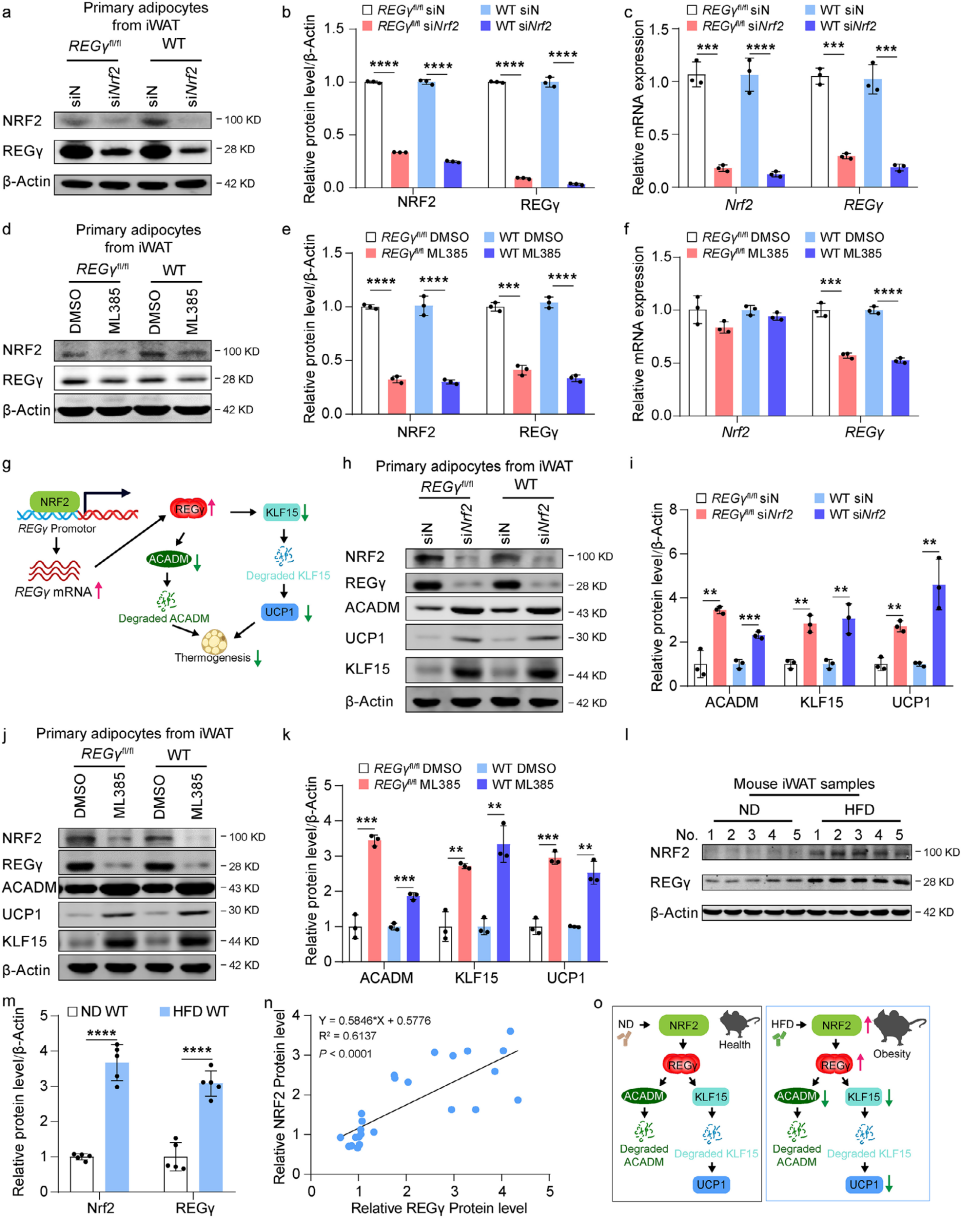
NRF2 regulates the transcription of REGγ to accelerate the process of obesity. a, b) Western blot analysis of NRF2, REGγ, and β‐Actin expression in primary adipocytes from 4‐week‐old *REGγ* ^fl/fl^ and WT mice with or without *Nrf2* knockdown (a). Quantification of NRF2 and REGγ protein expression (b). c) qRT‐PCR analysis of *REGγ* and *Nrf2* in primary adipocytes from 4‐week‐old *REGγ* ^fl/fl^ and WT mice with or without *Nrf2* knockdown. d, e) Western blot analysis of NRF2, REGγ, and β‐Actin expression in primary adipocytes from 4‐week‐old *REGγ* ^fl/fl^ and WT mice with or without NRF2 inhibitor (ML385 20 µM) treatment (d). Quantification of NRF2 and REGγ protein expression (e). f) qRT‐PCR analysis of *REGγ* and *Nrf2* in primary adipocytes from 4‐week‐old *REGγ* ^fl/fl^ and WT mice with or without NRF2 inhibitor (ML385 20 µM) treatment. g) Hypothetical schematic diagram illustrating the involvement of NRF2 in the regulation of REGγ expression. h, i) Western blot analysis of NRF2, REGγ, ACADM, KLF15, UCP1 and β‐Actin expression in primary adipocytes from 4‐week‐old *REGγ* ^fl/fl^ and WT mice with or without *Nrf2* knockdown (h). Quantification of ACADM, KLF15, and UCP1 protein expression (i). j, k) Western blot analysis of NRF2, REGγ, ACADM, KLF15, UCP1 and β‐Actin expression in primary adipocytes from 4‐week‐old *REGγ* ^fl/fl^ and WT mice with or without NRF2 inhibitor (ML385 20 µM) treatment (j). Quantification of ACADM, KLF15, and UCP1 protein expression (k). l, m) Western blot analysis of NRF2, REGγ, and β‐Actin expression in iWAT from ND‐ or HFD‐fed WT mice for 12 weeks (l). Quantification of NRF2 and REGγ protein expression (m). n) Correlation between the protein expression levels of NRF2 and REGγ in iWAT from the mice (*n* = 24). o) Schematic diagram illustrating the involvement of NRF2‐REGγ pathway and its downregulators (ACADM and KLF15) in regulating the process of Obesity. Statistical significance was assessed by unpaired Student's t test (b‐c, e‐f, i‐k, and m). Values were presented as Pearson's r correlation coefficient (n). *p < 0.05, **p < 0.01, ***p < 0.001, ****p < 0.0001.

In order to explore the molecular mechanism of the upstream regulation of REGγ, we first analyzed the impact of knocking down the reported transcription factors^[^ ^12^, ^28^, ^29^ ^]^ of REGγ and found that only Nrf2 regulates REGγ expression in primary adipocytes (Figure S8a,b, Supporting Information). As an important oxidative stress sensor, NRF2 plays a regulatory role in energy metabolism. An increasing amount of evidence suggests that NRF2 is a key target in obesity and related metabolic disorders.^[^ ^30^, ^31^, ^32^, ^33^, ^34^, ^35^ ^]^ Notably, NRF2 expression promotes lipid accumulation in adipocytes by increasing adipogenesis and decreasing lipolysis.^[^ ^35^ ^]^ We hypothesized that NRF2 may regulate browning of WAT through transcriptional regulation of REGγ. Based on these findings, we knocked down *Nrf2* in primary adipocytes from iWAT of *REGγ* ^fl/fl^ and WT mice and found that the mRNA and protein levels of *REGγ* were decreased (**Figure** **6**a–c). Conversely, overexpression of *Nrf2* in primary adipocytes from iWAT of *REGγ* ^fl/fl^ and WT mice resulted in increased REGγ protein expression levels (Figure S8c,d, Supporting Information). Moreover, treatment with NRF2 inhibitor ML385 in primary adipocytes from iWAT of *REGγ* ^fl/fl^ and WT mice inhibited REGγ expression (Figure 6d–f). Thus, NRF2 positively regulates the transcription of REGγ in browning of WAT (Figure 6g).

Next, we predicted the NRF2 sequence that binds to the REGγ promoter via the JASPAR database and identified two binding sites (Figure S8e,f, Supporting Information). We revealed that NRF2 directly binds to the REGγ promoter to regulate its transcription in primary adipocytes from iWAT of WT mice via ChIP‐qPCR assay (Figure S8g, Supporting Information), suggesting that NRF2 positively regulates the transcription of REGγ in browning of WAT.

Next, we knocked down *Nrf2* in primary adipocytes from iWAT of *REGγ* ^fl/fl^ and WT mice and found that the protein expression levels of ACADM, KLF15, and UCP1 were increased (Figure 6h,i). Conversely, overexpression of *Nrf2* in primary adipocytes from these mice resulted in downregulation of ACADM, KLF15, and UCP1 protein expression levels (Figure S8h, Supporting Information). Treatment with the NRF2 inhibitor ML385 in primary adipocytes upregulated ACADM, KLF15, and UCP1 protein expression (Figure 6j,k). Therefore, NRF2 positively regulates the transcription of REGγ and activates its downstream pathways to inhibit browning of WAT, thus promoting obesity.

Furthermore, we measured the protein expression of REGγ and NRF2 in iWAT from WT mice fed with either ND or HFD and found that the protein expression of REGγ and NRF2 was increased in iWAT from HFD‐fed WT mice than in ND‐fed mice (Figure 6l,m). In iWAT, the expression at the protein level of REGγ was positively correlated with NRF2 (Figure 6n). In iWAT from REGγ^fl/fl^ and WT mice fed with HFD compared with iWAT from mice fed with ND for 12 weeks, NRF2 and REGγ were increased, and ACADM, KLF15, and UCP1 were decreased (Figure S8i,j, Supporting Information).

In conclusion, the NRF2‐REGγ pathway regulates obesity through the browning of WAT (Figure 6o).

### 2.7 Knocking Down REGγ in iWAT Alleviates HFD‐Induced Obesity

**Figure 7:**
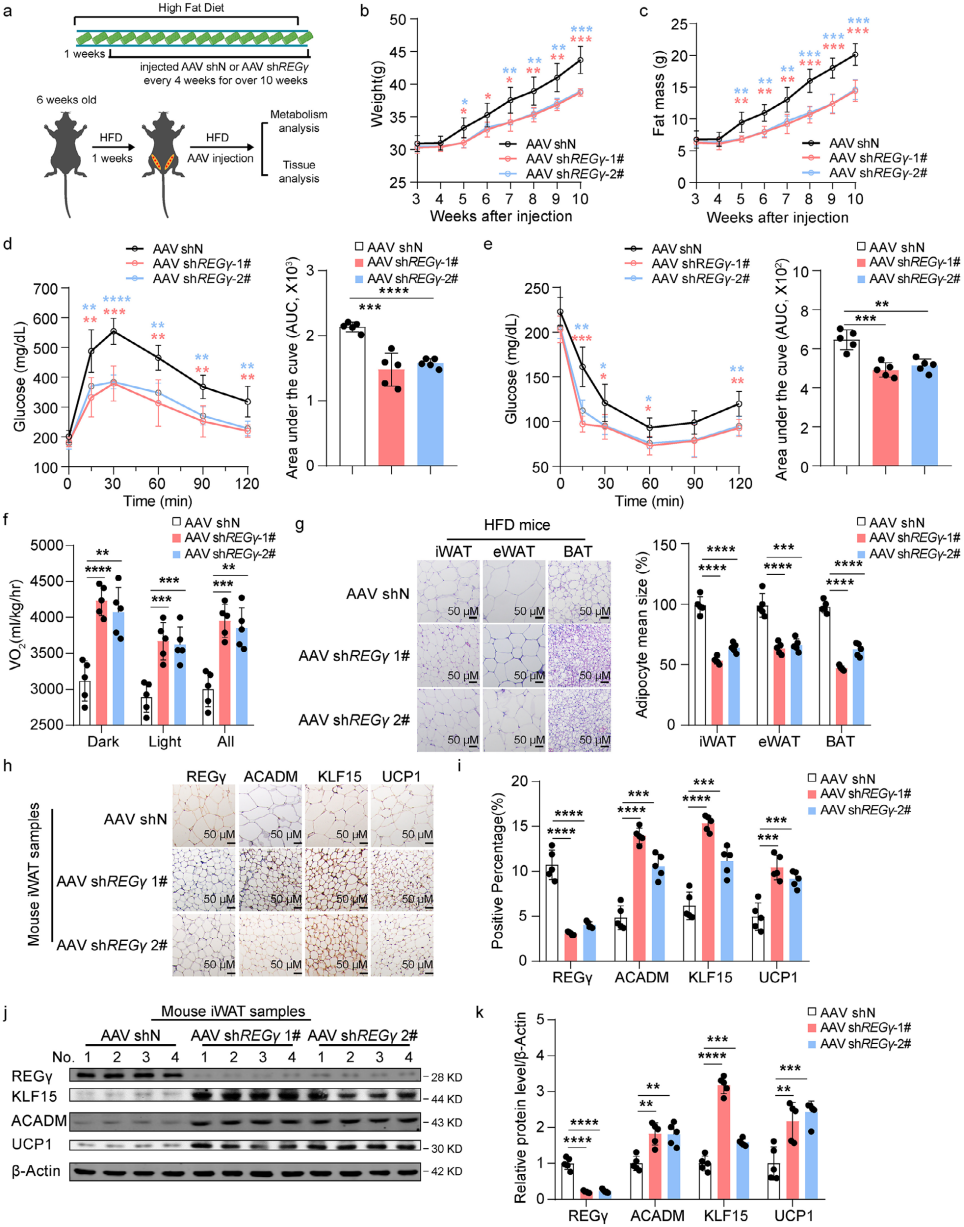
Inhibition of REGγ expression in iWAT alleviated HFD‐induced obesity. a) Schematic diagram of multipoint injection of inguinal fat pads of HFD‐fed mice injected with AAV shN, AAV sh*REGγ*‐1# or sh*REGγ*‐2# every 4 weeks for 11 weeks. b, c) Changes in body weight (b) and fat mass (c) of HFD‐fed mice subjected to AAV shN, AAV sh*REGγ*‐1# or sh*REGγ*‐2# injection over 11 weeks (*n* = 5). d) Glucose tolerance test in HFD‐fed mice subjected to AAV shN, AAV sh*REGγ*‐1# or sh*REGγ*‐2# injection for 12 weeks (*n* = 5). e) Insulin tolerance test in HFD‐fed mice subjected to AAV shN, AAV sh*REGγ*‐1# or sh*REGγ*‐2# injection for 12 weeks (*n* = 5). f) Whole‐body oxygen consumption analysis results of HFD‐fed mice injected with AAV shN, AAV sh*REGγ*‐1#, or sh*REGγ*‐2# injection for 6 weeks (*n* = 5). g) Representative H&E staining and quantification of iWAT, eWAT, and BAT from HFD‐fed mice subjected to AAV shN, AAV sh*REGγ*‐1#, or sh*REGγ*‐2# injection for 12 weeks (*n* = 5). Scale bar, 50 µm. h, i) IHC analysis of REGγ, ACADM, KLF15, and UCP1 expression in iWAT, eWAT, and BAT from HFD‐fed mice subjected to AAV shN, AAV sh*REGγ*‐1#, or sh*REGγ*‐2# injection for 12 weeks (h). Scale bar, 50 µm. Quantification of REGγ, ACADM, KLF15, and UCP1 protein expression (*n* = 5) (i). j, k) Western blot analysis of REGγ, ACADM, KLF15, and UCP1 expression in iWAT, eWAT, and BAT from HFD‐fed mice injected with AAV shN, AAV sh*REGγ*‐1#, or sh*REGγ*‐2# for 12 weeks (j). Scale bar, 50 µm. Quantification of REGγ, ACADM, KLF15, and UCP1 protein expression (*n* = 5) (k). Statistical significance was assessed by two‐way ANOVA (b‐f), or unpaired Student's t test (g, i, and k). *p<0.05, **p < 0.01, ***p < 0.001, ****p < 0.0001.

To explore whether downregulating REGγ in iWAT alleviates obesity induced by an HFD diet and to provide new insights for obesity treatment, we inhibited REGγ expression via AAV shREGγ GFP or AAV shN GFP administration in the iWAT of WT mice (**Figure** **7**a). We discovered that the inhibition of REGγ expression in WT mice resulted in a smaller body size, reduced body fat, improved glucose tolerance, and increased insulin sensitivity after being fed a HFD for 10 weeks (Figure 7b–e). Metabolic cage experiment revealed increased O_2_ consumption in mice injected with AAV *shREGγ* compared with AAV shN feeding on HFD for 6 weeks (Figure 7f). We subsequently observed significantly lower volumes and weights of BAT, iWAT, and eWAT in mice injected with AAV *shREGγ* than AAV shN feeding on HFD for 10 weeks (Figure 7g; Figure S9a–c, Supporting Information). The protein levels of ACADM, KLF15, and UCP1 were increased after knocking down REGγ in iWAT, and were accompanied by decreased liver weights and lipid infiltration (Figure 7h–k; Figure S9d–f, Supporting Information). Moreover, TG, TC, and LDL levels were decreased, and HDL levels were increased in the serum of the mice injected with AAV *shREGγ* compared with AAV shN feeding on HFD for 10 weeks (Figure S9g, Supporting Information). Using qRT‒PCR, we subsequently examined the mRNA levels of *REGγ* and thermogenic genes (*Ucp1* and *Pgc‐1α*) in iWAT from mice injected with AAV *shREGγ* compared with AAV shN feeding on HFD for 10 weeks. We detected increased mRNA levels of thermogenic genes (*Ucp1* and *Pgc‐1α*) (Figure S9h, Supporting Information). Overall, these results demonstrate that REGγ regulates the browning of white adipose tissue via ACADM and KLF15‐UCP1 signaling pathways to modulate obesity, providing new insights for the treatment of obese patients.

## 3 Discussion

In this study, we discovered that REGγ expression was significantly upregulated in adipose tissue of obese individuals and in the iWAT tissue of obese mice. Suppressing REGγ expression in vivo reduced fat deposition, increased energy expenditure in adipose tissue, and protected mice from HFD‐induced obesity and insulin resistance. Obesity manifests as excessive fat accumulation and is the result of dysfunction in lipid metabolism,^[^ ^2^ ^]^ thus to reduce obesity it requires interventions are required related to both energy intake and expenditure. More importantly, active brown and beige fat represents the crux of investigations into energy metabolism.^[^ ^5^, ^36^, ^37^, ^38^ ^]^ In both humans and mice, the activation of brown and beige fat results in increased insulin sensitivity and glucose tolerance.^[^ ^6^, ^39^, ^40^, ^41^ ^]^ Browning of white adipose tissue promotes weight loss, improves insulin resistance, and corrects hyperlipidemia associated with obesity, suggesting its potential in managing obesity and its complications.^[^ ^9^ ^]^ Interestingly, we also reported that the REGγ knockout mouse developed a smaller body size.^[^ ^11^ ^]^ These findings strongly suggest that REGγ is involved in the regulation of browning of white adipose tissue to modulate obesity.

Owing to its high mitochondrial content, BAT is brown or reddish‐brown and dissipates energy.^[^ ^42^, ^43^, ^44^ ^]^ More importantly, active brown and beige fat represent the crux of investigations into energy metabolism.^[^ ^5^, ^36^, ^37^, ^38^ ^]^ And the activation of brown and beige fat results in increased insulin sensitivity and glucose tolerance.^[^ ^6^, ^39^, ^40^, ^41^ ^]^ The important biological functions of the non‐classical REGγ proteasome pathway in many physiological and pathological processes.^[^ ^11^, ^12^, ^13^, ^14^, ^15^, ^16^, ^17^, ^18^, ^19^, ^20^, ^21^, ^22^, ^23^, ^45^, ^46^ ^]^ Our previous studies have revealed that REGγ gene deletion protects against HFD‐induced hepatic steatosis in mice by inhibiting Sirt1‐mediated cellular autophagy and thereby affecting the development of HFD‐induced hepatic steatosis.^[^ ^23^ ^]^ Mechanistically, we had found that REGγ regulated browning of WAT by modulating ACADM signaling in an ubiquitin‐independent degradation manner to influence obesity. In our study, we found no significant change in transcription of *Acadm*, a significantly increased protein in the WAT of the deletion of REGγ. Mechanistically, REGγ regulated browning of WAT by degrading ACADM in an ubiquitin‐independent degradation manner. Suppression of ACADM could increase the levels of triglycerides, phospholipids, and LDs.^[^ ^47^ ^]^ We observed that suppression or deletion of REGγ decreased TG, TC, and LDL levels, and increased the HDL level feeding on ND or HFD in mice. These results signified that REGγ could regulate browning of WAT by degrading ACADM in an ubiquitin‐independent degradation manner to inhibit browning of WAT.

Brown adipocytes express a specific protein in their mitochondrial inner membrane, UCP1, which converts the energy from glucose and fatty acid breakdown into heat by preventing ATP formation, increasing body heat production.^[^ ^6^ ^]^ The increased expression of UCP1 in WAT leads to energy expenditure and reduced lipid accumulation within adipocytes, resembling classic brown adipocyte functions. The unique thermogenic capacity of brown and beige fat is attributable to the high density of mitochondria and the expression of UCP1 in these thermogenic adipocytes.^[^ ^7^, ^8^ ^]^ Beige adipose tissue represents an intermediate type of adipose tissue found within WAT. Beige adipocytes, which arise from white adipocyte transformation or de novo differentiation from progenitor cells,^[^ ^48^ ^]^ share similar characteristics with brown adipocytes, including multiple small lipid droplets and dense mitochondria, and express key thermogenic genes (*Ucp1*, *Cidea*, and *Pgc‐1α*).^[^ ^5^, ^6^ ^]^ Interestingly, in our study, we found that deletion of REGγ significantly upregulated UCP1 in iWAT tissue. However, there is no research on the regulation of thermogenesis and catabolism by REGγ. Furthermore, we have discovered in the mechanism that REGγ regulates KLF15‐UCP1 signaling in an ubiquitin‐independent degradation manner to modulate browning of WAT, which is a new mechanism for regulating UCP1. These findings strongly suggest that REGγ is a key regulator of metabolic processes associated with obesity. Thus, we conducted in vivo therapeutic experiments of iWAT with in‐situ multi‐point inhibition of REGγ, and found that knocking down REGγ by AAV‐shREGγ in iWAT alleviates HFD‐induced obesity, indicating that REGγ could be a good target to alleviate obesity. Of course, the shortcomings (or future) are that we need to find inhibitors of REGγ‐proteasome to further explore the treatment of obesity.

Existing studies have consistently shown that oxidative stress is markedly elevated in the WAT of both HFD‐induced and genetically obese models, as well as in obese humans. As a consequence, the expression and activity of NRF2 are upregulated, exacerbating lipid accumulation and promoting obesity.^[^ ^35^ ^]^ NRF2 also emerges as a pivotal regulator of WAT browning and energy metabolism, making it a critical target in obesity and related metabolic disorders.^[^ ^31^, ^32^, ^49^, ^50^, ^51^ ^]^ Our previous research has confirmed that NRF2 directly regulates REGγ mRNA expression.^[^ ^12^ ^]^ In this study, we found that the NRF2‐REGγ axis facilitates adipose tissue dysfunction, contributing to obesity. Based on these observations, we propose that the NRF2‐REGγ axis is not only integral to the regulation of WAT browning but also holds significant potential as a therapeutic target for combating obesity‐related metabolic diseases.

## 4 Experimental Section

### Animal Model

All animal experiments involved in this study were approved by the Ethics Committee of Animal Experiments of East China Normal University (m20210604). REGγ knockout mice were kindly provided by Dr. John J. Monaco at the University of Cincinnati.^[^ ^52^ ^]^ Mice with a targeted deletion of *REGγ* in adipose tissues (*REGγ* ^fl/fl^ *Adipoq*‐cre) were generated by crossing the *REGγ* ^fl/fl^ mice with transgenic mice expressing Cre recombinase under the control of the adiponectin promoter (*Adipoq*‐cre). Mice from the same litter that did not express Cre were used as the control group. The mice were treated with a high‐fat diet (60%, ResearchDiet, D12492) for 10–12 weeks to establish an obesity model (DIO model).^[^ ^53^, ^54^ ^]^

### Human Patients

The subcutaneous white adipose tissues (sWATs) from obese individuals (*n* = 5, body mass index (BMI) **≥** 30) and the sWATs from normal weight individuals (*n* = 5, 18 **≤** BMI < 25) were obtained from Shanghai Fifth People's Hospital. The experimental study was approved by the Ethics Committee of Shanghai Fifth People's Hospital affiliated to Fudan University (Ethics No.149, 2024). All participants in this study provided written informed consent.

### GTT, ITT, and Cold Tolerance Test

For GTT,^[^ ^53^ ^]^ the mice were fasted overnight before the glucose tolerance test (GTT). Glucose (1.25 g kg^−1^ body weight) was injected intraperitoneally, and blood glucose levels were measured at 0, 15, 30, 60, and 120 min after injection. For ITT,^[^ ^53^ ^]^ the mice were fasted for 6 h before the insulin tolerance test (ITT) and were injected with insulin (1.25 or 1.5 U kg^−1^ body weight), and their blood glucose levels were measured at 0, 15, 30, 60, and 120 min after injection. We used the area under the curve (AUC) for assessing insulin and glucose tolerance.^[^ ^54^ ^]^ For cold exposure, mice were housed at 4 °C, and core temperature was measured at the indicated time.^[^ ^54^ ^]^

### Primary Mouse Preadipocytes

Primary mouse preadipocytes were isolated from 4‐week‐old mice. After cervical dislocation, the samples were soaked in 75% alcohol for 5 min. The adipose tissue was separated under sterile conditions, cut into 1 mm^3^ tissue fragments, and 1 mL of tissue digestion solution (20 mg of type II collagenase + 10 mL PBS+100 µL 1 M HEPES)^[^ ^55^ ^]^ was added, and the samples were digested in a 37 °C water bath for 30 min until there were no large tissue fragments. The mixture was centrifuged at 2400 rpm for 10 min, and the supernatant was discarded. After it was resuspended in DMEM, the mixture was filtered through a 40 µm filter and centrifuged at 2400 rpm for 10 min before the supernatant was discarded. After the cells were resuspended in DMEM (DMEM supplemented with 10% fetal bovine serum and 1% penicillin/streptomycin), they were placed in a culture dish at 37 °C with 5% CO_2_. Once the cells had reached the wall, the DMEM was replaced, and the culture was continued.

### Differentiated Adipocytes

To induce the differentiation and maturation of adipocytes after cell attachment, the cells were cultured in induction medium containing 6 µg mL^−1^ insulin, 0.5 mM IBMX, 50 nM T3, 1 µM dexamethasone, and 1 µM rosiglitazone for 2 days, followed by the addition of maintenance medium containing 6 µg mL^−1^ insulin, 50 nM T3, and 1 µM rosiglitazone for 4 days. The medium was changed every 2 days, and mature adipocytes were induced on the 6th day.

### Immunohistochemistry

The dissected adipose tissues and liver tissues were fixed with 4% paraformaldehyde for 48 h, embedded in paraffin, and cut into 5 µm sections. After tissue sectioning and baking, the samples were dewaxed with xylene and hydrated with gradient alcohol. Hematoxylin and eosin (HE) staining was used for morphometric analysis of adipocyte size. After gradient dewaxing and dehydration, IHC staining was performed, and the samples were repaired with citric acid repair solution for 20 min, followed by treatment with 3% hydrogen peroxide for 10 min to block endogenous peroxidase activity. After blocking with 5% BSA, the sections were incubated with the primary antibody overnight at 4 °C. On the second day, the sections were incubated with the secondary antibody at room temperature for 30 min and stained with DAB to observe the degree of staining. Afterward, the samples were stained with hematoxylin and subjected to dehydration with an ethanol gradient before being sealed. Images were captured with an optical microscope (Nikon). The adipocyte size was quantified via ImageJ.

### Western Blot Analysis

Cells or tissues were lysed using RIPA buffer containing 50 mM Tris‐HCl (pH 7.5), 150 mM NaCl, 1 mM EDTA, phosphatase inhibitor, and protease inhibitor to prepare protein samples, and electrophoresis was performed in a 10%–12% SDS polyacrylamide gel. After transfer to a membrane, the samples were incubated overnight at 4 °C with primary antibodies against REGγ (Abcam, ab157157, 1:2000), NRF2 (CST, 12721S, 1:2000), ACADM (Abcam, ab92461, 1:2000), KLF15 (Santa Cruz Biotechnology, sc‐271675, 1:2000), UCP1 (Proteintech, 23673‐1‐AP, 1:2000), and β‐actin (Proteintech, 66009‐1‐Ig, 1:5000) at 4 °C. Subsequent incubation with a fluorescence‐labeled secondary antibody (1:5000 dilution) enabled precise detection of specific protein signals using a LI‐COR Odyssey infrared imaging system.

### Co‐Immunoprecipitation Assays

The cell precipitates were collected, RIPA buffer (50 mM Tris HCl, 150 mM NaCl, 1 mM EDTA, 10% glycerol, and protease inhibitor) was added, and the mixture was incubated on ice for 30 min. The mixture was subsequently centrifuged, and the supernatant was collected. Ten percent total protein was collected as input and stored at −20 °C. Simultaneously, anti‐DYKDDDDK/anti‐HA agarose and protein lysis mixture were incubated overnight at 4 °C, and protein expression was determined via SDS‒PAGE.

### qRT‐PCR

Total RNA was extracted from adipose tissues or cells via TRIzol (TAKARA). Then, PrimeScriptTM RT Master Mix (TaKaRa, RR036A) was used to reverse transcribe the RNA into cDNA. Real‐time fluorescence quantitative PCR was performed using SYBR Green PCR Master Mix (Vazyme, Q711‐02) on a Roche LightCycler 480 (Roche). The experiment was repeated three times, and gene expression was measured via the 2^−ΔΔCt^ method. The primers used in these studies are listed in Table S1 (Supporting Information).

### Local Delivery of Adeno‐Associated Virus (AAV) in Adipose Tissues

Adeno‐associated virus (AAV) vector‐mediated overexpression of mouse shRNAs (targeting REGγ and the scrambled control) was constructed, amplified, and purified by Hanbio Biotechnology (Shanghai, China). A total of 50 µL of 1×10^9^ Vg µL^−1^ of each type of AAV diluted in PBS was injected into the inguinal fat pads of the mice, with each fat pad injected four times. The changes in body weight, fat content, and oxygen consumption of the mice were monitored, the mice were euthanized, and their tissues were dissected for further analysis.^[^ ^54^ ^]^

### Body Composition Analysis and Metabolic Cage Experiments

The mice were fed a normal diet (11% kcal fat, LabDiet, 5053) or an HFD (60% kcal fat, ResearchDiet, D12492) for the indicated times. The systemic compositions of the mice were measured via an AccuFat MRI system (AccuFat‐1050, MAG‐MED) to measure body fat and lean mass accurately. The mice were free to eat and drink in a Comprehensive Lab Animal Monitoring System (CLAMS, Columbus Instruments) metabolic cage system, and their oxygen consumption, food intake, and physical activity were measured. The mice were individually housed for 72 h, and data were collected during light and dark cycles.

### Cycloheximide (CHX) Assay for Protein Stability

To measure protein stability, adipocytes were seeded in a 6‐well plate until the density reached 70%. Then, 100 µg mL^−1^ cyclohexide (Chx, MCE) was added to block protein synthesis. The cells were collected at specific time points, and protein expression was determined via Western blotting.

### In Vitro Proteolytic Analysis

The ACADM protein obtained through in vitro translation and a TNT Quick Coupled Transcription/Translation System (Promega) was used for in vitro translation. One µg of purified REGγ heptamer and 0.25 µg of purified 20S proteasome were reacted at 30 °C for different durations. Further analysis was conducted through protein blotting.

### ChIP‐qPCR

Immortalized adipocytes were prepared and cross‐linked at room temperature with a final concentration of 1% formaldehyde for 10 min, after which cross‐linking with glycine was terminated. The cells were washed with precooled PBS, lysis buffer (1% SDS, 10 mM EDTA, protease inhibitor, and 50 mM Tris HCl (pH 8.1)), and sonicated. After centrifugation, the lysate was diluted in ChIP dilution buffer (0.01% SDS, 1.0% Triton X‐100, 1.2 mM EDTA, 16.7 mM NaCl, protease inhibitor, and 16.7 mM Tris HCl (pH 8.1)). Then, a ChIP anti‐NRF2 antibody (CST) or IgG (CST) was used for immunoprecipitation using magnetic beads, which were subsequently incubated overnight at 4 °C. Afterward, the sample was washed and crosslinked at 65 °C for over 6 h. The DNA was then purified and subjected to qPCR analysis using specific primers.

### Statistical Analysis

Statistical analysis was performed via GraphPad Prism 8 software. All the data are expressed as the means ± standard deviations (SDs). For two independent datasets, a two‐tailed unpaired Student's t‐test was used. For multiple comparisons, one‐way or two‐way analysis of variance (ANOVA) was used, followed by a Holm–Sidak post hoc test. All experiments were repeated at least three times or performed with independent experiments or biological samples, unless stated specifically, and representative data are shown. NS, *p *> 0.05. *, *p *< 0.05. **, *p *< 0.01. ***, *p *< 0.001. ****, *p *< 0.0001.

## Conflict of Interest

The authors declare no conflict of interest.

## Author Contributions

H.C., Q.G., and S.G. contributed equally to this work. L.L. and H.C. conceived and designed the research. H.C., Q.G., and S.G. performed most of the biochemical and molecular experiments and bioinformatics analysis, with assistance from Y.D., Z.Z., Y.L., L.L., and B.X. Y.G., H.C., Q.G., and S.G. performed in vivo experiments. H.C., L.Z., Z.Z., T.P., H.Z., G.Y., and S.Y. contributed to the clinical sample collection. L.L., H.C., Q.G., and Z.Z. edited the manuscript. L.L., H.C., and Q.G. wrote the manuscript.

## Supporting information



Supporting Information

## Data Availability

The data that support the findings of this study are available from the corresponding author upon reasonable request.
