# Supplementary material for: NRF2‐REGγ‐ACADM/KLF15 Signaling Pathway Regulates the Browning of White Adipose Tissue to Modulate Obesity
Source: Adv Sci (Weinh). 2025 Sep 19;12(46):e09429. doi: 10.1002/advs.202509429 (PMC12697771; doi:10.1002/advs.202509429)
Supplement: Supplementary file 1 — Supporting Information [file ADVS-12-e09429-s001.docx]

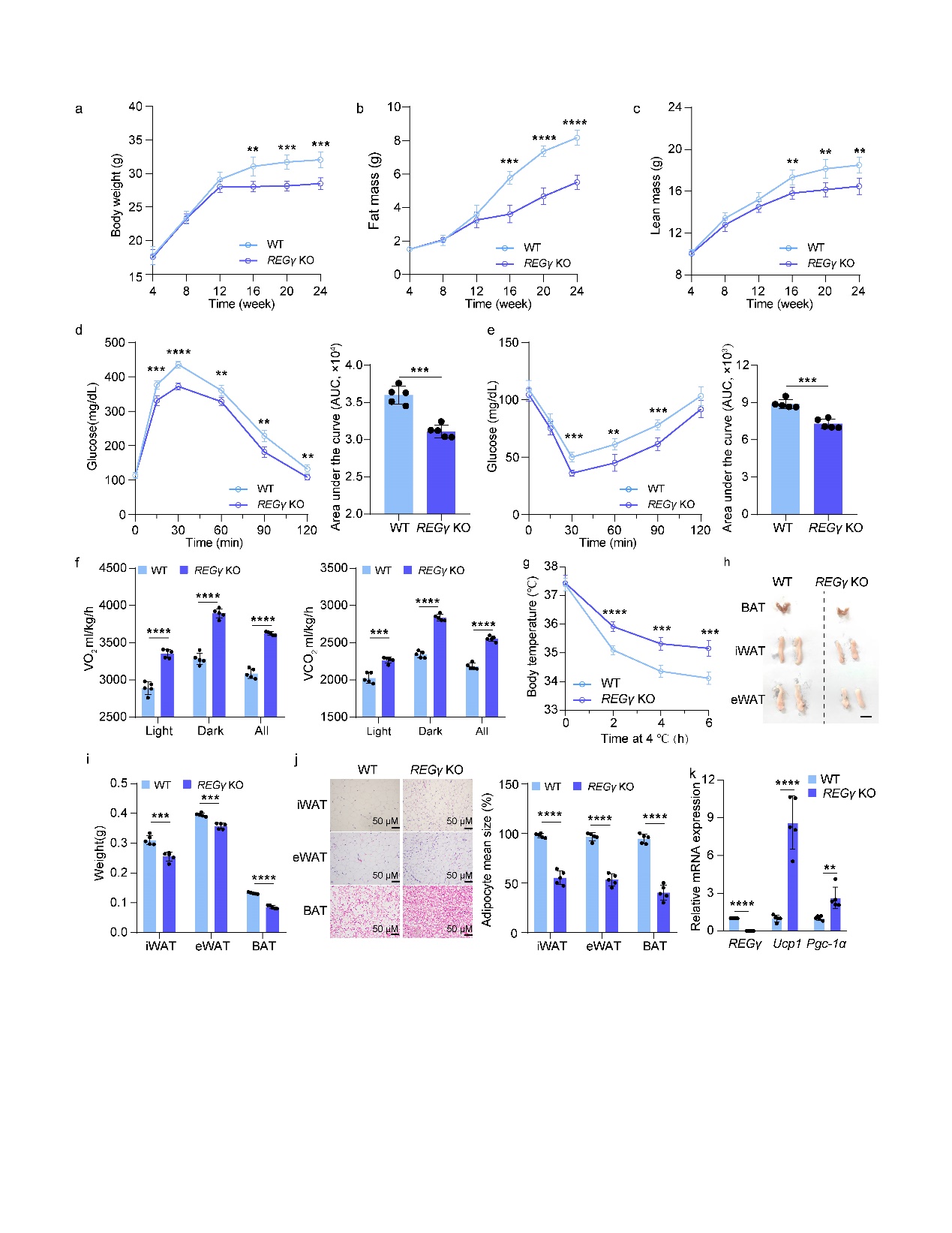


**Figure S1** REGγ deficiency reduced fat deposition and increased energy expenditure in whole-body knockout mice. a-c) Changes in body weight (a), fat mass (b) and lean mass (c) in 4-week-old to 24-week-old wildtype (WT) and *REGγ* knockout (*REGγ* KO) mice (*n* = 5). d) Glucose tolerance test in 24-week-old WT and *REGγ* KO mice (*n* = 5). e) Insulin tolerance test in 24-week-old WT and *REGγ* KO mice (*n* = 5). f) Whole-body oxygen consumption and carbon dioxide consumption analysis results of 24-week-old WT and *REGγ* KO mice (*n* = 5). g) Cold tolerance analysis of 24-week-old WT and *REGγ* KO mice (*n* = 5). h) Representative images of BAT, eWAT and iWAT from 24-week-old WT and *REGγ* KO mice (*n* = 5). Scale bar, 1 cm. i) Weights of iWAT, eWAT and BAT from 24-week-old WT and *REGγ* KO mice (*n* = 5). j) Representative H&E staining and quantification of iWAT, eWAT and BAT from 24-week-old WT and *REGγ* KO mice (*n* = 5). Scale bar, 50 µm. k) qRT-PCR analysis of expression of *REGγ* and thermogenic genes (*Ucp1* and *Pgc-1α*) in iWAT from 24-week-old WT and *REGγ* KO mice (*n* = 5). Statistical significance was assessed by two-way ANOVA (a-e and g), or unpaired Student’s t test (f, i, j and k). *p<0.05, **p<0.01, ***p<0.001, ****p<0.0001


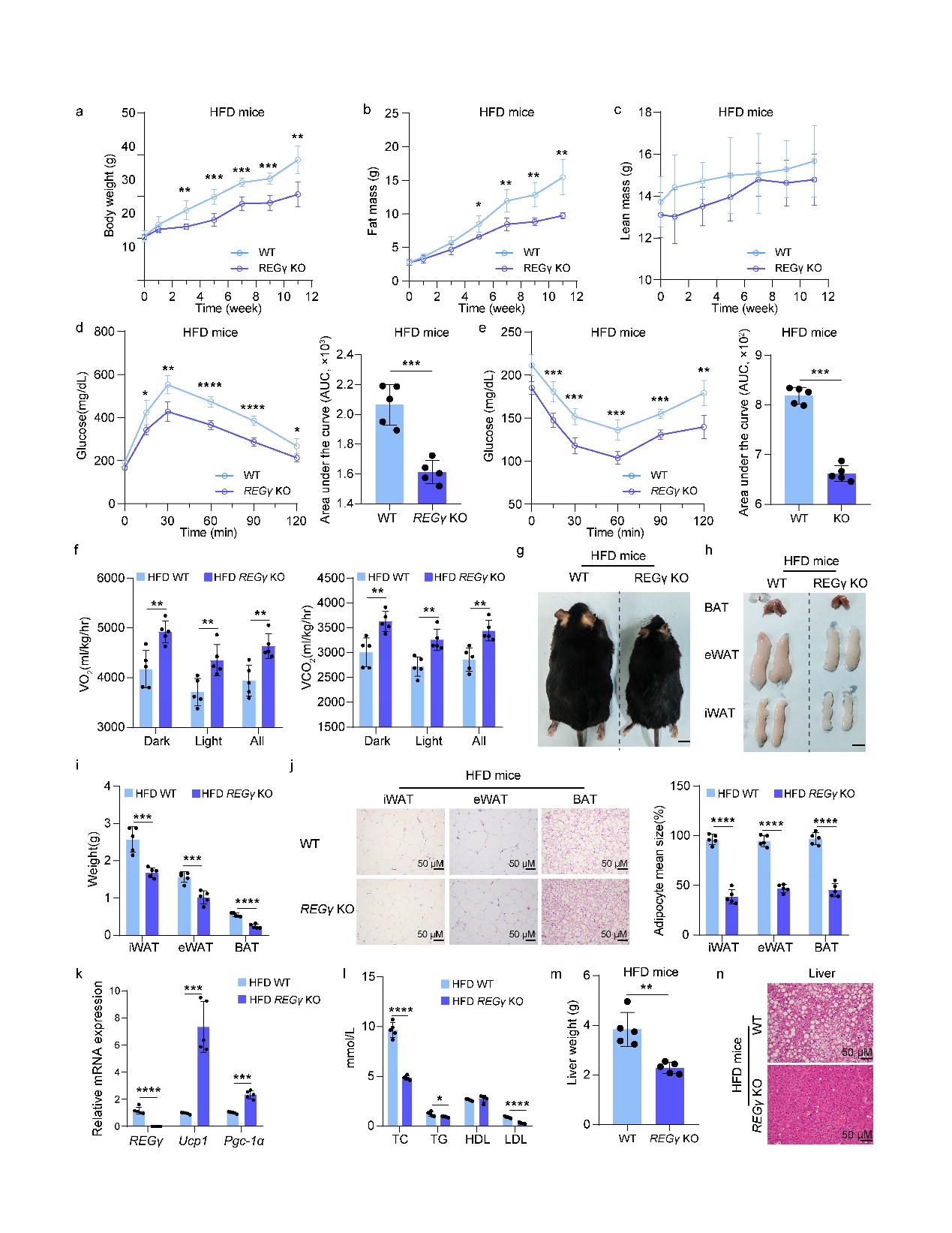


**Figure S2.** Deficiency of REGγ expression protects mice from high-fat-diet-induced obesity and insulin resistance. a-c) Changes in body weight (a), fat mass (b) and lean mass (c) in WT and *REGγ* KO mice fed a HFD (60%, ResearchDiet, D12492) for 12 weeks (*n* = 5). d) Glucose tolerance test in in WT and *REGγ* KO mice fed a HFD for 12 weeks (*n* = 5). e) Insulin tolerance test in WT and *REGγ* KO mice fed a HFD for 12 weeks (*n* = 5). f) Whole-body oxygen consumption and carbon dioxide consumption analysis results of WT and *REGγ* KO mice fed a HFD for 4 weeks (*n* = 5). g) Representative images of WT and *REGγ* KO mice fed a HFD for 12 weeks (*n* = 5). h) Representative images of BAT, eWAT and iWAT from WT and *REGγ* KO mice fed a HFD for 12 weeks (*n* = 5). Scale bar, 1 cm. i) Weights of iWAT, eWAT and BAT from WT and *REGγ* KO mice fed a HFD for 12 weeks (*n* = 5). j) Representative H&E staining and quantification of iWAT, eWAT and BAT from WT and *REGγ* KO mice fed a HFD for 12 weeks (*n* = 5). Scale bar, 50 µm. k) qRT-PCR analysis of expression of *REGγ* and thermogenic genes (*Ucp1* and *Pgc-1α*) in iWAT from WT and *REGγ* KO mice fed a HFD for 12 weeks (*n* = 5). l) ELISA analysis of blood lipids, including TC, TG, HDL, and LDL in the serum of WT and *REGγ* KO mice fed a HFD for 12 weeks (*n* = 5). m) Weights of livers in WT and *REGγ* KO mice fed a HFD for 12 weeks (*n* = 5). n) Representative H&E staining of livers from WT and *REGγ* KO mice fed a HFD for 12 weeks (*n* = 5). Statistical significance was assessed by two-way ANOVA (a-e), or unpaired Student’s t test (f, i-l and m). *p<0.05, **p<0.01, ***p<0.001, ****p<0.0001.


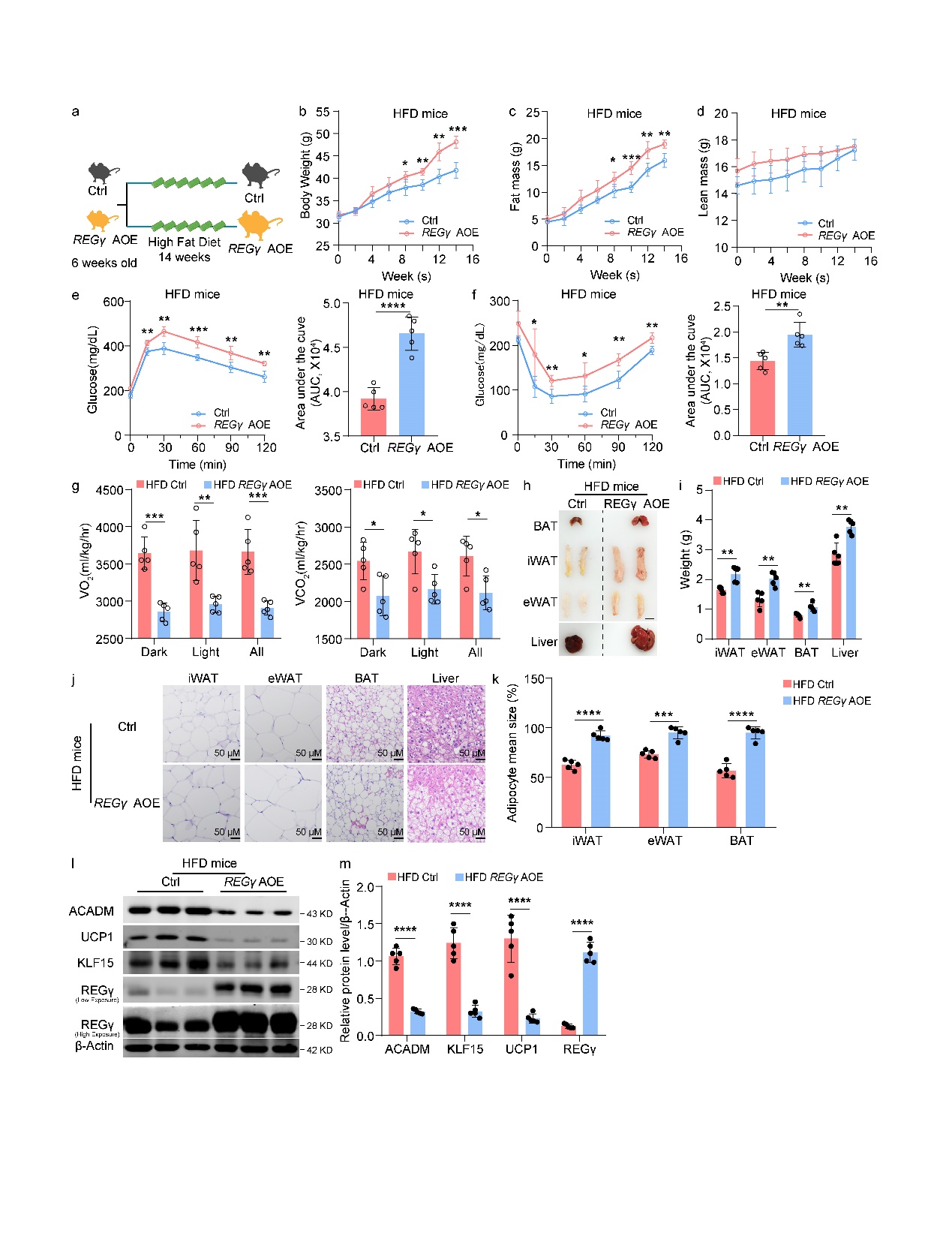


**Figure S3.** Overexpression of REGγ promotes high-fat-diet-induced obesity and insulin resistance in mice. a) Schematic diagram of multipoint injection of inguinal fat pads of HFD-fed Ctrl and *REGγ* AOE for 14 weeks. b-d) Changes in body weight (b), fat mass (c) and lean mass (d) in Ctrl and *REGγ* AOE mice fed a HFD (60%, ResearchDiet, D12492) for 14 weeks (*n* = 5). e) Glucose tolerance test in HFD-fed Ctrl and *REGγ* AOE mice for 14 weeks (*n* = 5). f) Insulin tolerance test in HFD-fed Ctrl and *REGγ* AOE mice for 14 weeks (*n* = 5). g) Whole-body oxygen consumption analysis results in Ctrl and *REGγ* AOE mice fed HFD for 6 weeks (*n* = 5). h) Representative images of BAT, eWAT, iWAT and liver from HFD-fed Ctrl and *REGγ* AOE for 14 weeks (*n* = 5). Scale bar, 1 cm. i) Weights of BAT, eWAT, iWAT and liver from HFD-fed Ctrl and *REGγ* AOE for 14 weeks (*n* = 5). j) Representative H&E staining of iWAT, eWAT, BAT and liver in HFD-fed Ctrl and *REGγ* AOE mice for 14 weeks (*n* = 5). Scale bar, 50 µm. k) Quantification of adipocyte mean size in BAT, eWAT, and iWAT from HFD-fed Ctrl and *REGγ* AOE mice for 14 weeks (*n* = 5). l-m) Western blot analysis of REGγ, ACADM, KLF15 and UCP1 expression in iWAT from HFD-fed Ctrl and *REGγ* AOE mice for 14 weeks (*n* = 5) (l). Scale bar, 50 µm. Quantification of REGγ, ACADM, KLF15 and UCP1 protein expression (m). Statistical significance was assessed by two-way ANOVA (b-f), or unpaired Student’s t test (g, i-k and m). *p<0.05, **p<0.01, ***p<0.001, ****p<0.0001.


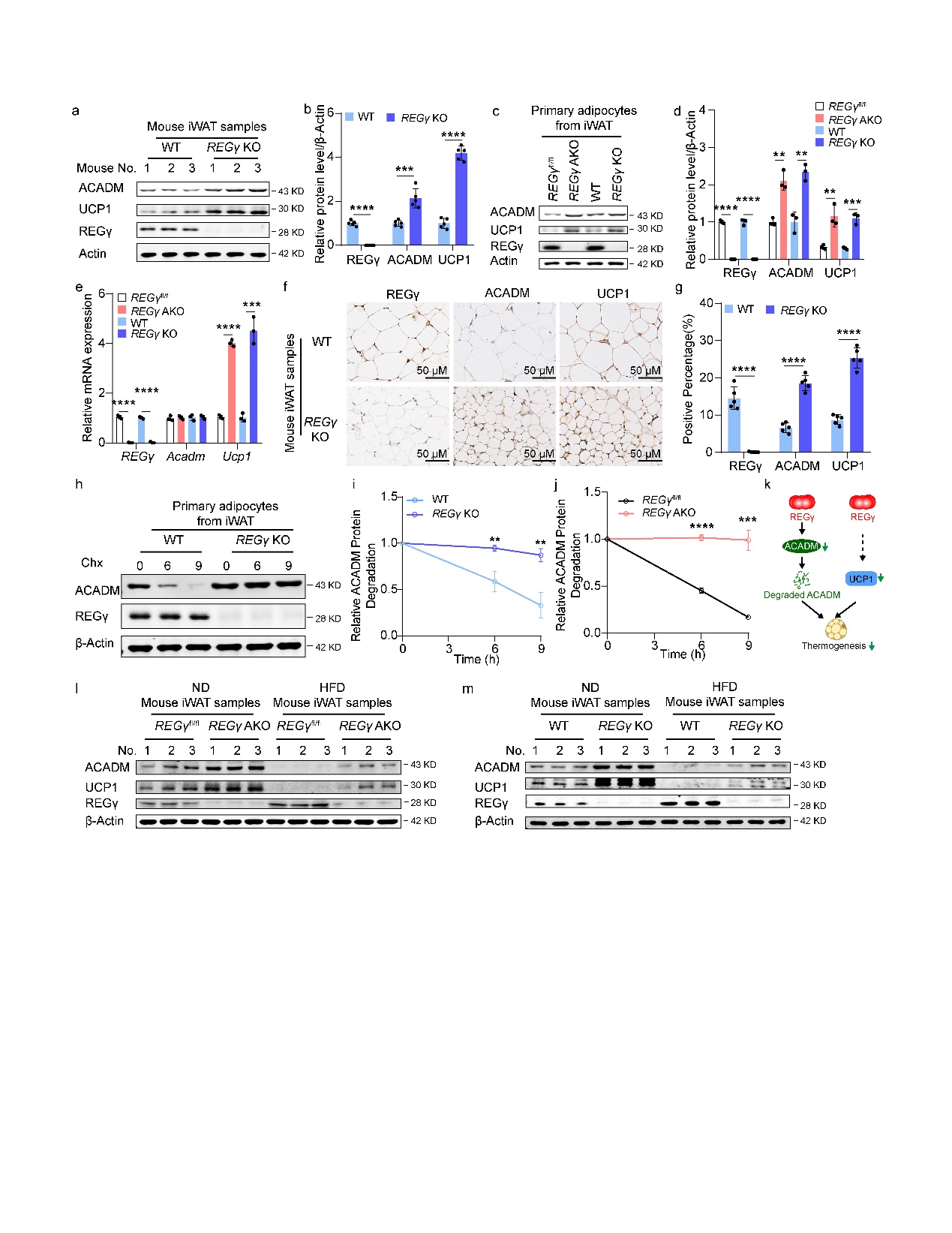


**Figure S4.** REGγ regulates ACADM expression via ubiquitin-independent degradation to induce obesity. a-b) Western blot analysis of REGγ, UCP1, ACADM and β-Actin expression in iWAT from 24-week-old WT and *REGγ* KO mice (a). Quantification of REGγ, UCP1 and ACADM protein expression (b) (*n* = 5). c-d) Western blot analysis of REGγ, UCP1, ACADM and β-Actin expression in primary adipocytes from 4-week-old *REGγ*^fl/fl^, *REGγ* AKO, WT and *REGγ* KO mice (c). Quantification of REGγ, UCP1 and ACADM protein expression (d). e) qRT-PCR analysis of *REGγ*, *Acadm* and *Ucp1* expression in primary adipocytes from 4-week-old *REGγ*^fl/fl^, *REGγ* AKO, WT and *REGγ* KO mice (*n* = 5). f-g) IHC analysis of REGγ, UCP1 and ACADM in iWAT from 24-week-old WT and *REGγ* KO mice (f). Quantification of REGγ, UCP1 and ACADM protein expression (g) (*n* = 5). Scale bar, 50 µm. h-i) Western blot analysis of ACADM, REGγ and β-Actin expression in primary adipocytes from 4-week-old WT and *REGγ* KO mice after 0, 6 h or 9 h of Cycloheximide (Chx) treatment (h). Quantification of degradation (i). j) Western blot analysis of REGγ, ACADM and β-Actin after the degradation of ACADM in vitro. k) Hypothetical schematic diagram illustrating the involvement of REGγ in the regulation of thermogenesis. l) Western blot analysis of ACADM, UCP1, REGγ and β-Actin expression in iWAT from ND- or HFD- fed *REGγ*^fl/fl^ and *REGγ* AKO mice (*n* = 5). m) Western blot analysis of ACADM, UCP1, REGγ and β-Actin expression in iWAT from ND- or HFD-fed WT and *REGγ* KO mice (*n* = 5). Statistical significance was assessed by unpaired Student’s t test (b, d, e, g and i). *p<0.05, **p<0.01, ***p<0.001, ****p<0.0001.


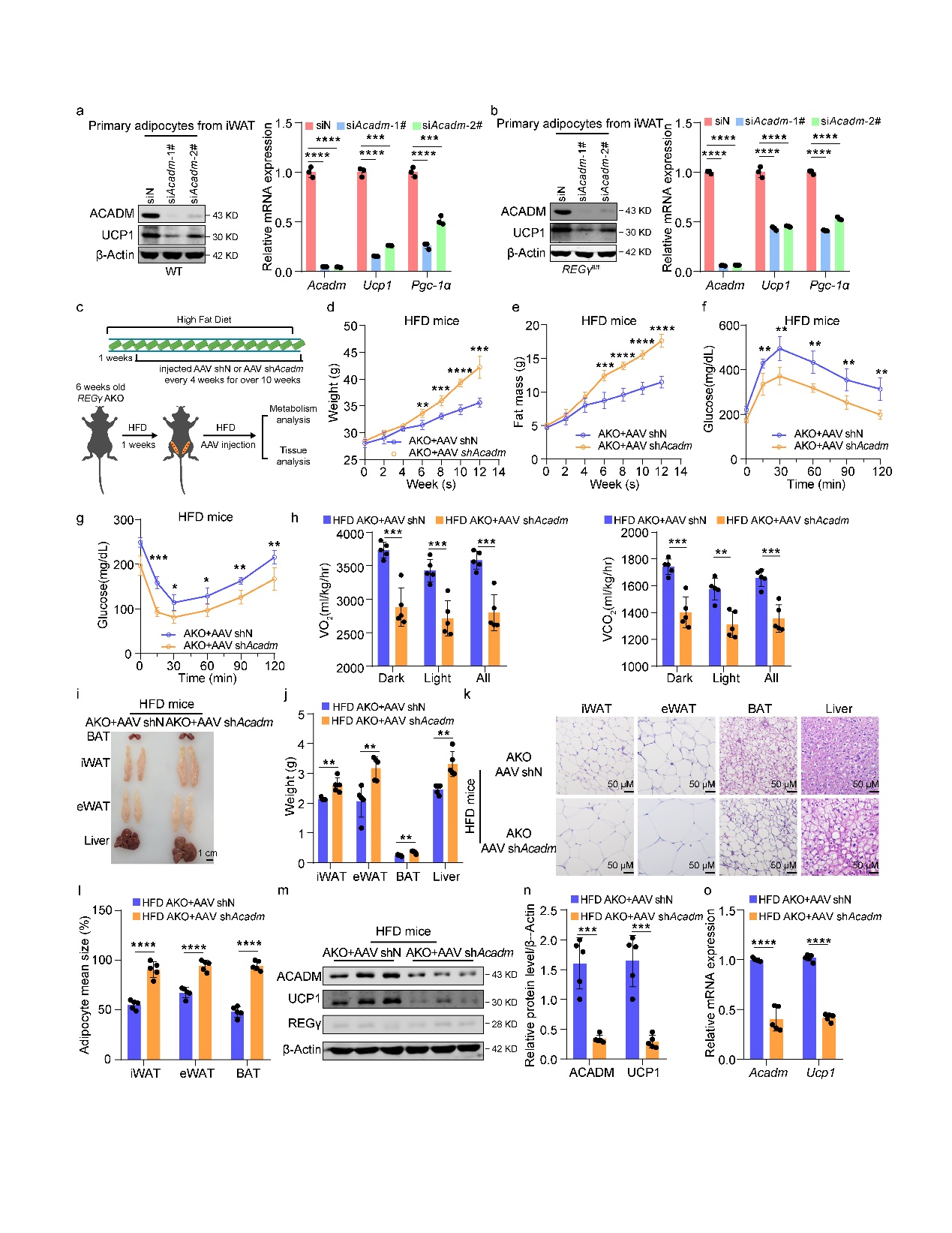


**Figure S5.** REGγ regulates the browning of white adipose tissue by degrading ACADM. a-b) Western blot analysis of ACADM, UCP1 and β-Actin expression in primary adipocytes from 4-week-old WT and *REGγ*^fl/fl^ mice with or without *Acadm* knockdown and qRT-PCR analysis of *Acadm，*thermogenic genes (*Ucp1* and *Pgc-1α*) in primary adipocytes from 4-week-old WT and *REGγ*^fl/fl^ mice with or without *Acadm* knockdown. c) Schematic diagram of multipoint injection of inguinal fat pads of HFD-fed *REGγ* AKO mice injected with AAV shN or AAV sh*Acadm* every 4 weeks for 10 weeks. d-e) Changes in body weight (d) and fat mass (e) of HFD-fed *REGγ* AKO mice subjected to AAV shN or AAV sh*Acadm* injection for 12 weeks (*n* = 5). f) Glucose tolerance test in HFD-fed *REGγ* AKO mice subjected to AAV shN or AAV sh*Acadm* injection for 12 weeks (*n* = 5). g) Insulin tolerance test in HFD-fed *REGγ* AKO mice subjected to AAV shN or AAV sh*Acadm* injection for 12 weeks (*n* = 5). h) Whole-body oxygen consumption analysis results of HFD-fed *REGγ* AKO mice injected with AAV shN or AAV sh*Acadm* injection for 6 weeks (*n* = 5). i) Representative images of BAT, eWAT, iWAT and liver from HFD-fed *REGγ* AKO mice subjected to AAV shN or AAV sh*Acadm* injection for 12 weeks (*n* = 5). Scale bar, 1 cm. j) Weights of BAT, eWAT, iWAT and liver from HFD-fed *REGγ* AKO mice subjected to AAV shN or AAV sh*Acadm* injection for 12 weeks (*n* = 5). k) Representative haematoxylin and eosin (H&E) staining in BAT, eWAT, iWAT and liver from HFD-fed *REGγ* AKO mice subjected to AAV shN or AAV sh*Acadm* injection for 12 weeks (*n* = 5). Scale bar, 50 µm. l) Quantification of adipocyte mean size in BAT, eWAT, and iWAT from HFD-fed *REGγ* AKO mice subjected to AAV shN or AAV sh*Acadm* injection for 12 weeks (*n* = 5). m) Western blot analysis of ACADM, UCP1, REGγ and β-Actin expression in iWAT from HFD-fed *REGγ* AKO mice subjected to AAV shN or AAV sh*Acadm* injection for 12 weeks (*n* = 5). n) Quantification of ACADM and UCP1 protein expression (*n* = 5). o) qRT-PCR analysis of *Acadm*，thermogenic genes (*Ucp1*) in iWAT from HFD-fed *REGγ* AKO mice subjected to AAV shN or AAV sh*Acadm* injection for 12 weeks (*n* = 5). Statistical significance was assessed by two-way ANOVA (d-g), or unpaired Student’s t test (a, b, h, j, l, n and o). *p<0.05, **p<0.01, ***p<0.001, ****p<0.0001. *REGγ*^fl/fl^ *Adipoq*-cre, AKO.


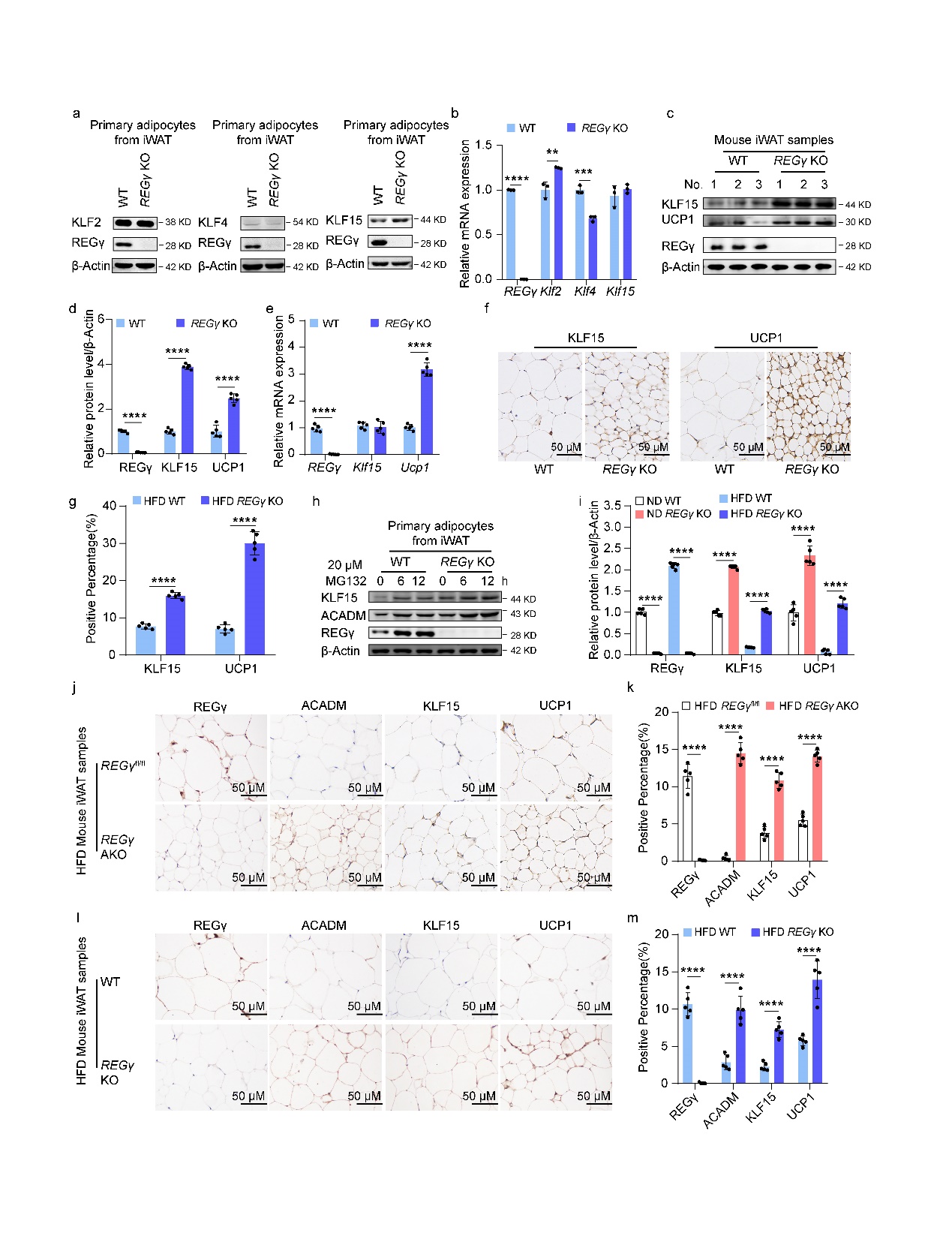


**Figure S6.** REGγ modulates KLF15 expression via ubiquitin-independent degradation to induce obesity. a) Western blot analysis of KLF2, KLF4, KLF15 and β-Actin expression in primary adipocytes from 4-week-old WT and *REGγ* KO mice. b) qRT-PCR analysis of KLF2, KLF4, KLF15 and 18S expression in primary adipocytes from 4-week-old WT and *REGγ* KO mice. c-d) Western blot analysis of REGγ, UCP1, KLF15 and β-Actin expression in iWAT from 24-week-old WT and *REGγ* KO mice (c). Quantification of REGγ, UCP1 and KLF15 protein expression (d) (*n* = 5). e) qRT-PCR analysis of REGγ, KLF15 and UCP1 expression in iWAT from 24-week-old WT and *REGγ* KO mice (*n* = 5). f-g) IHC analysis of KLF15 and UCP1 expression in iWAT from from 24-week-old WT and *REGγ* KO mice (f). Scale bar, 50 µm. Quantification of KLF15 and UCP1 protein expression (g) (*n* = 5). h) Western blot analysis of KLF15, UCP1, REGγ and β-Actin expression in primary adipocytes from 4-week-old WT and *REGγ* KO mice after 0, 6 h or 12 h of MG132 treatment. i) Quantification the protein expression of KLF15, UCP1, REGγ and β-Actin in iWAT from ND or HFD fed WT and *REGγ* KO mice for 12 weeks (*n* = 5). Related to Figure 5o. j-k) IHC analysis of REGγ, ACADM, KLF15 and UCP1 expression in iWAT from HFD fed *REGγ*^fl/fl^ and *REGγ* AKO mice for 12 weeks (j). Scale bar, 50 µm. Quantification of REGγ, ACADM, KLF15 and UCP1 protein expression (k) (*n* = 5). l-m) IHC analysis of REGγ, ACADM, KLF15 and UCP1 expression in iWAT from HFD-fed WT and *REGγ* KO mice for 12 weeks (l). Scale bar, 50 µm. Quantification of REGγ, ACADM, KLF15 and UCP1 protein expression (m) (*n* = 5). Statistical significance was assessed by unpaired Student’s t test (b, d, e, g, i, k and m). *p<0.05, **p<0.01, ***p<0.001, ****p<0.0001.


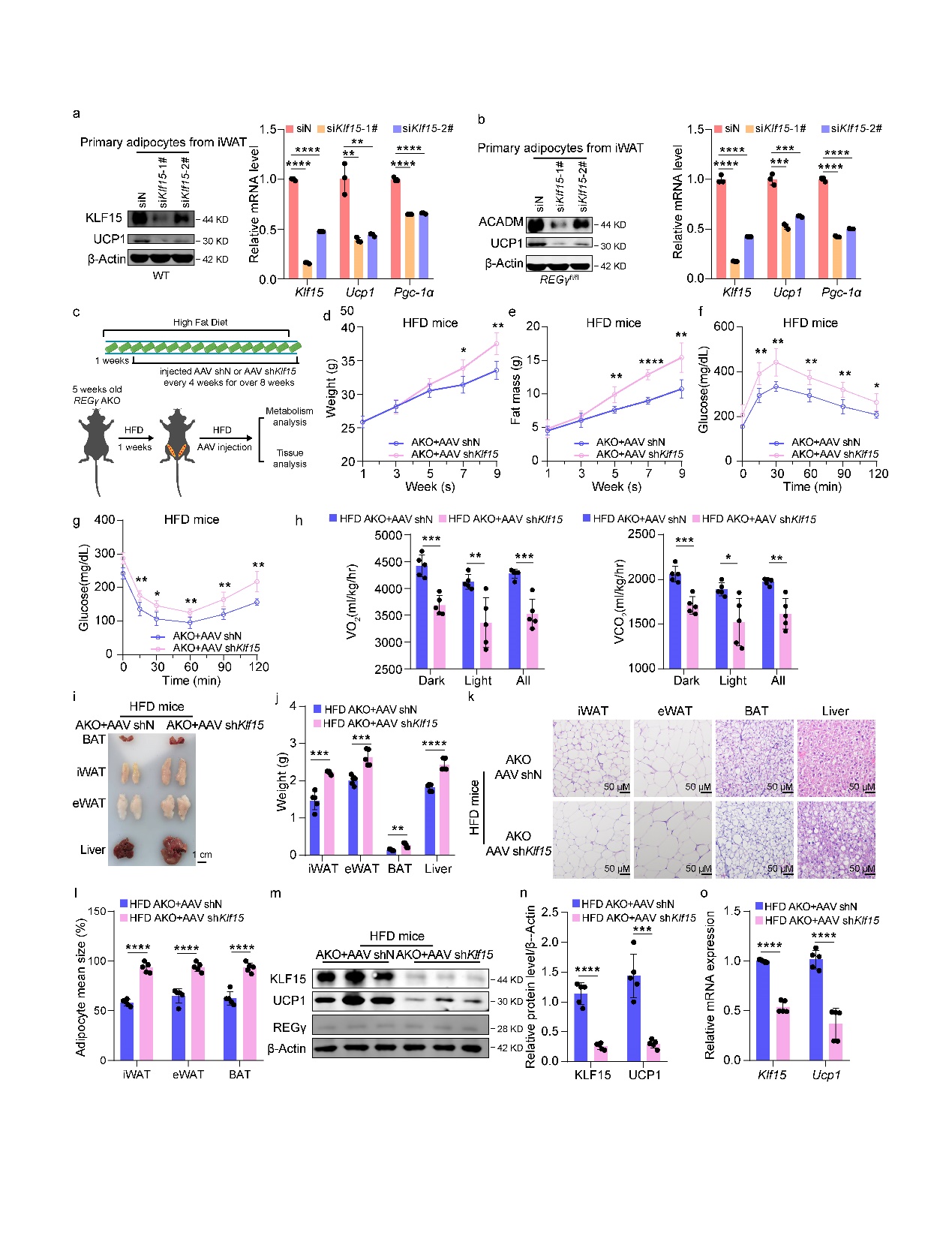


**Figure S7.** REGγ regulates the browning of white adipose tissue by degrading KLF15. a-b) Western blot analysis of KLF15, UCP1 and β-Actin expression in primary adipocytes from 4-week-old WT and *REGγ*^fl/fl^ mice with or without *Klf15* knockdown and qRT-PCR analysis of *Klf15*, thermogenic genes (*Ucp1* and *Pgc-1α*) in primary adipocytes from 4-week-old WT and *REGγ*^fl/fl^ mice with or without *Klf15* knockdown. c) Schematic diagram of multipoint injection of inguinal fat pads of HFD-fed *REGγ* AKO mice injected with AAV shN and AAV sh*Klf15* every 4 weeks for 8 weeks. d-e) Changes in body weight (d) and fat mass (e) of HFD-fed *REGγ* AKO mice subjected to AAV shN or AAV sh*Klf15* injection for 9 weeks (*n* = 5). f) Glucose tolerance test in HFD-fed *REGγ* AKO mice subjected to AAV shN or AAV sh*Klf15* injection for 9 weeks (*n* = 5). g) Insulin tolerance test in HFD-fed *REGγ* AKO mice subjected to AAV shN or AAV sh*Klf15* injection for 9 weeks (*n* = 5). h) Whole-body oxygen consumption analysis results of HFD-fed *REGγ* AKO mice injected with AAV shN or AAV sh*Klf15* injection for 6 weeks (*n* = 5). i) Representative images of BAT, eWAT, iWAT and liver from HFD-fed *REGγ* AKO mice subjected to AAV shN or AAV sh*Klf15* injection for 9 weeks (*n* = 5). Scale bar, 1 cm. j）Weights of BAT, eWAT, iWAT and liver from HFD-fed *REGγ* AKO mice subjected to AAV shN or AAV sh*Klf15* injection for 9 weeks (*n* = 5). k) Representative haematoxylin and eosin (H&E) staining in BAT, eWAT, iWAT and liver from HFD-fed *REGγ* AKO mice subjected to AAV shN or AAV sh*Klf15* injection for 9 weeks (*n* = 5). Scale bar, 50 µm. l) Quantification of adipocyte mean size in BAT, eWAT, and iWAT from HFD-fed *REGγ* AKO mice subjected to AAV shN or AAV sh*Klf15* injection for 9 weeks (*n* = 5). m) Western blot analysis of KLF15, UCP1, REGγ and β-Actin expression in iWAT from HFD-fed *REGγ* AKO mice subjected to AAV shN or AAV sh*Klf15* injection for 9 weeks (*n* = 5). n) Quantification of KLF15, and UCP1 protein expression (*n* = 5). o) qRT-PCR analysis of *Klf15*, thermogenic genes (*Ucp1*) in iWAT from HFD-fed *REGγ* AKO mice subjected to AAV shN or AAV sh*Klf15* injection for 9 weeks (*n* = 5). Statistical significance was assessed by two-way ANOVA (d-g), or unpaired Student’s t test (a, b, h, j, l, n and o). *p<0.05, **p<0.01, ***p<0.001, ****p<0.0001. *REGγ*^fl/fl^ *Adipoq*-cre, AKO.


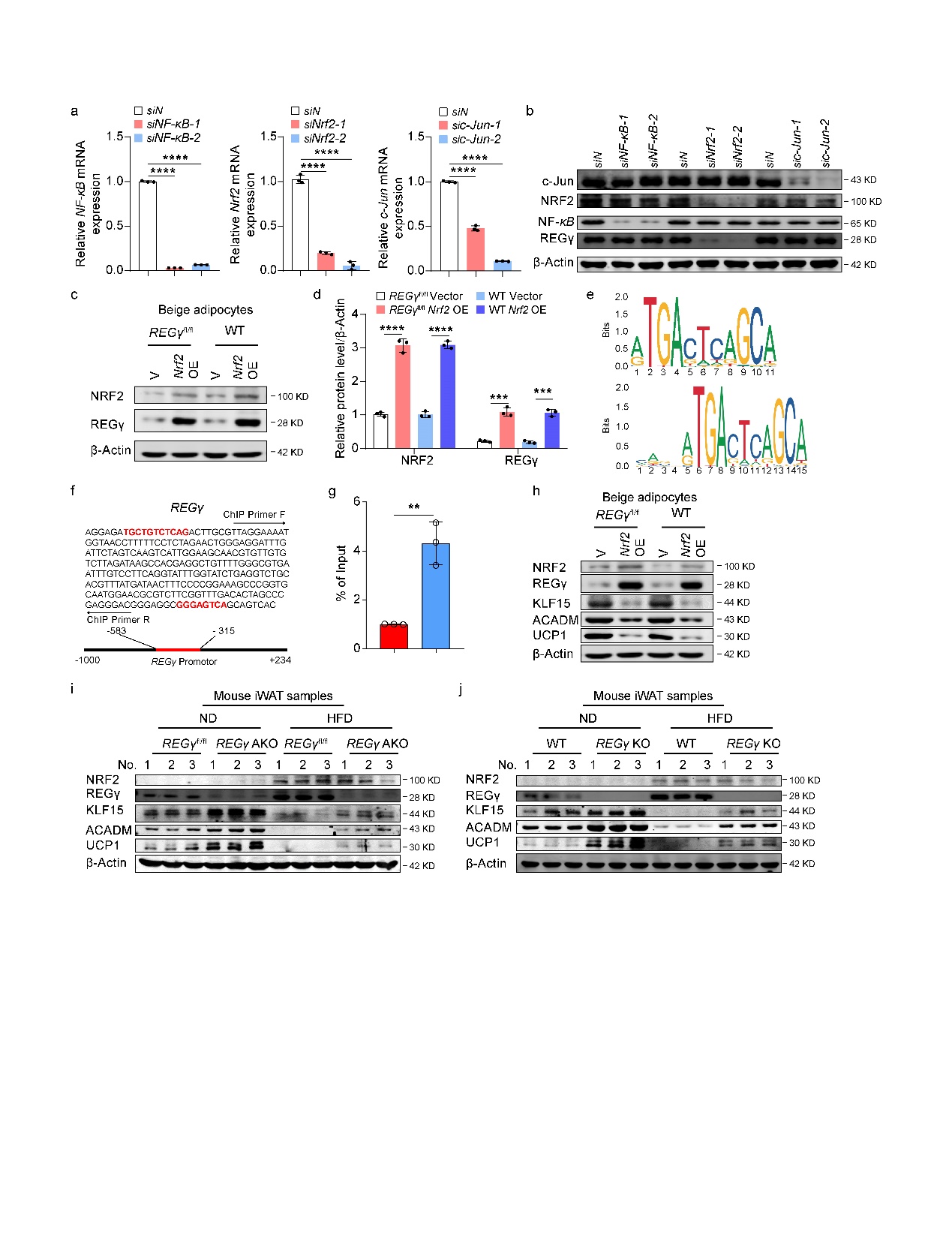


**Figure S8.** Nrf2 regulates the transcription expression of REGγ to accelerate the process of obesity. a) qRT-PCR analysis of *NF-κB*, *Nrf2*, *c-Jun* and *18S* expression in primary adipocytes from 4-week-old WT mice with or without si*NF-κB*, *Nrf2* or *c-Jun*. b) Western blot analysis of REGγ, NF-κB, NRF2, c-Jun, and β-Actin expression in primary adipocytes from 4-week-old WT with or without si*NF-κB*, si*Nrf2* or si*c-Jun*. c-d) Western blot analysis of NRF2, REGγ and β-Actin expression in primary adipocytes from 4-week-old *REGγ*^fl/fl^ and WT mice with or without NRF2 overexpression (c). Quantification of NRF2 and REGγ protein expression (d). e-f) Sequence of NRF2 binding to *REGγ* promoter as predicted by the JASPAR database (e). *REGγ* ChIP-qPCR primer sequences and binding sites (red) (f). g) ChIP-qPCR analysis of the interaction between NRF2 and REGγ in primary adipocytes from 4-week-old *REGγ*^fl/fl^ mice. h) Western blot analysis of NRF2, REGγ, ACADM, KLF15, UCP1 and β-Actin expression in primary adipocytes from 4-week-old *REGγ*^fl/fl^ and WT mice with or without NRF2 overexpression. i) Western blot analysis of NRF2, REGγ, KLF15, ACADM, UCP1 and β-Actin in iWAT from ND- or HFD-fed *REGγ*^fl/fl^ and *REGγ* AKO mice for 16 weeks (*n* = 5). j) Western blot analysis of NRF2, REGγ, KLF15, ACADM, UCP1 and β-Actin expression in iWAT from ND- or HFD-fed WT and *REGγ* KO mice for 16 weeks (*n* = 5). Statistical significance was assessed by unpaired Student’s t test (a, d and g). *p<0.05, **p<0.01, ***p<0.001, ****p<0.0001.


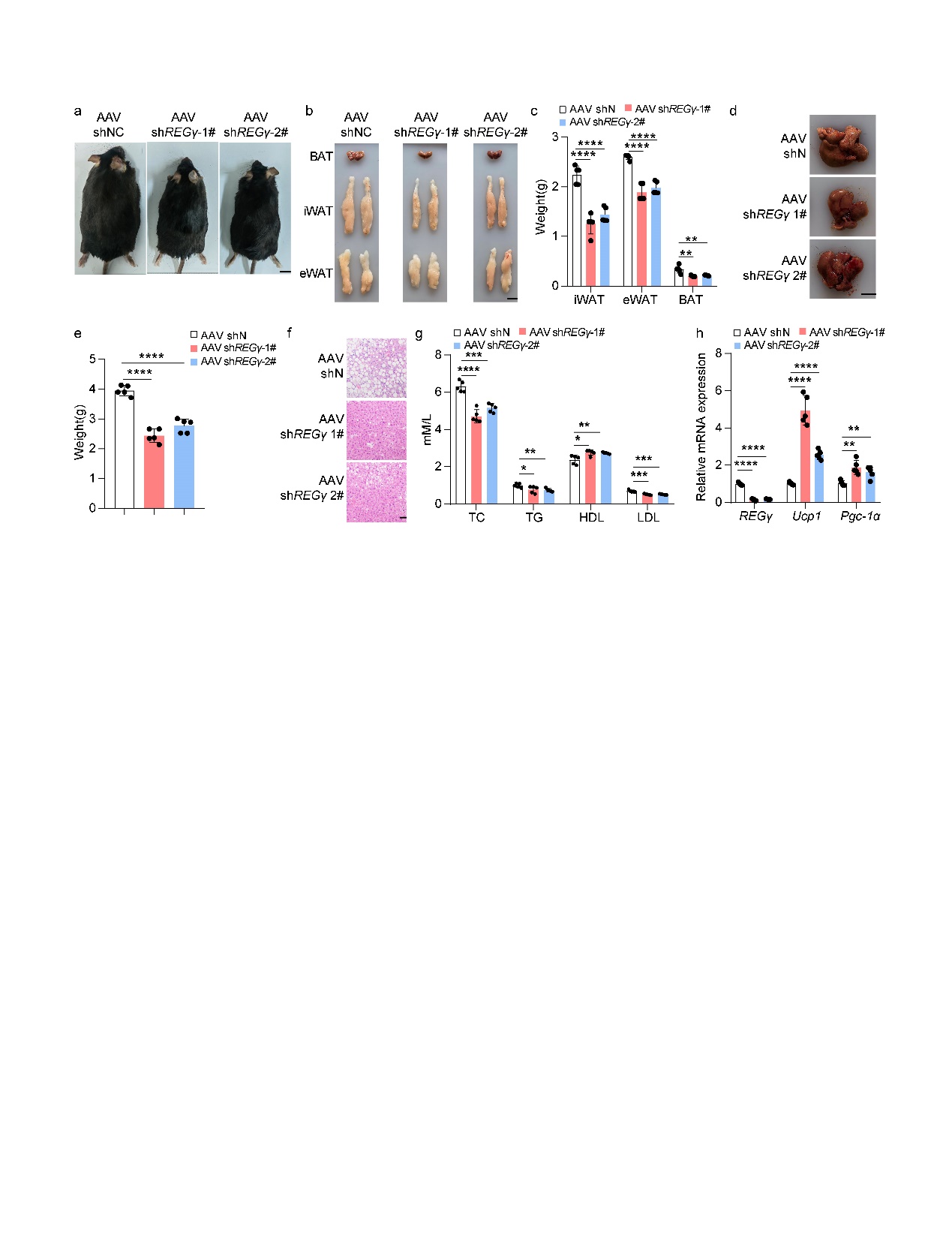


**Figure S9.** Inhibition of REGγ expression in the iWAT alleviated high-fat diet induced obesity. a) Representative images of HFD-fed mice injected with AAV shN, AAV sh*REGγ*-1# or sh*REGγ*-2# for 11 weeks (*n* = 5). b) Representative images of BAT, eWAT and iWAT from HFD-fed mice subjected to AAV shN, AAV sh*REGγ*-1# or sh*REGγ*-2# injection for 12 weeks (*n* = 5). Scale bar, 1 cm. c) Weights of iWAT, eWAT and BAT from HFD-fed mice subjected to AAV shN, AAV sh*REGγ*-1# or sh*REGγ*-2# injection for 11 weeks (*n* = 5). d) Representative images of liver from HFD-fed mice subjected to AAV shN, AAV sh*REGγ*-1# or sh*REGγ*-2# injection for 12 weeks (*n* = 5). e) Liver weights of HFD-fed mice subjected to AAV shN, AAV sh*REGγ*-1# or sh*REGγ*-2# injection for 12 weeks (*n* = 5). f) Representative images of liver from HFD-fed mice subjected to AAV shN, AAV sh*REGγ*-1# or sh*REGγ*-2# injection for 12 weeks (*n* = 5). Scale bar, 50 μm. g) ELISA analysis of blood lipids, including TC, TG, HDL, and LDL, in the serum of HFD-fed mice injected with AAV shN, AAV sh*REGγ*-1# or sh*REGγ*-2# for 12 weeks (*n* = 5). h) qRT‒PCR analysis of expression of *REGγ* and thermogenic genes (*Ucp1* and *Pgc-1α*) genes in iWAT from HFD-fed mice injected with AAV shN, AAV sh*REGγ*-1# or sh*REGγ*-2# for 12 weeks (*n* = 5). Statistical significance was assessed by unpaired Student’s t test (c, e, g and h). *p<0.05, **p<0.01, ***p<0.001, ****p<0.0001.

**Supplementary Table 1**

| **Primers for qPCR** | | |
| --- | --- | --- |
| Gene | Forward Primer sequence | Reverse Primer sequence |
| *18s* | GGACACGGACAGGATTGACA | GACATCTAAGGGCATCACAG |
| *m-REGγ* | CCCACTTACAAGAAGCGCAG | CAGCTGAACCCACATTTTGA |
| *m-Nrf2* | CAGCATAGAGCAGGACATGGAG | GAACAGCGGTAGTATCAGCCAG |
| *m-Acadm* | AGGATGACGGAGCAGCCAATGA | GCCGTTGATAACATACTCGTCAC |
| *m-Klf15* | GCAGTGGAGGTATTGGAGATAG | AGAAATTCAGGGAAGCAGAAATG |
| *m-Klf2* | CACCTAAAGGCGCATCTGCGTA | GTGACCTGTGTGCTTTCGGTAG |
| m-*Klf4* | CTATGCAGGCTGTGGCAAAACC | TTGCGGTAGTGCCTGGTCAGTT |
| m-*Ucp1* | GGCCCTTGTAAACAACAAAATAC | GGCAACAAGAGCTGACAGTAAAT |
| m-*Pgc1α* | ACCATGACTACTGTCAGTCACTC | GTCACAGGAGGCATCTTTGAAG |
| **Primers for Genotype Identification** | | |
| Genotype | Primer name | Primer sequence |
| *REGγ* WT/KO | Common | CACGATGGACTGGATGGT |
|  | Wildtype | CTAACATAACTTACCTTGCC |
|  | Knockout | TCGAGCGAGCACGTACT |
| *REGγ*^fl/fl^ | Psme3-loxP1 | AATTTCAAGGTGAGGGCGAGACAG |
|  |  | AATAAACGTGGGACAGTCCCTCACT |
|  | Psme3-loxP2 | ACCAGCTCAGGAGGTAAGGTCCAAT |
|  |  | CACAATTCTAAGTGACTCCACCCCC |
| Cre | cre700-F | GCCGCATTACCGGTCGATGCAAGA |
|  | cre700-R | GTGGCAGATGGCGCGGCAACACCATT |
| *LSL-REGγ* | OE1 | CAGACTTGTGGGATACAGAAGAC |
|  | OE2 | AGTCCACCTCACTCCTCATAAC |
|  | OE3 | GGTTGGCTATAAAGAGGTCATCAG |

**Supplementary Table 2**

| **REAGENT or RESOURCE** | **SOURCE** | **IDENTIFIER** |
| --- | --- | --- |
| **Antibody** | | |
| Mouse-anti-β-Actin antibody | MBL International | Cat# M177-3; |
| Mouse-anti-α-Tubulin antibody | Cell Signaling Technology | Cat# 3873； |
| Rabbit-anti-PSME3 antibody | Abcam | Cat# ab157157; |
| Rabbit-anti-ACADM antibody | Cell Signaling Technology | Cat# ab92461; |
| Rabbit-anti-KLF15 antibody | Santa Cruz Biotechnology | Cat# sc-271675; |
| Rabbit-anti-NRF2 antibody | Cell Signaling Technology | Cat# 12721S; |
| Rabbit-anti-UCP1 antibody | Proteintech | Cat# 23673-1-AP; |
| Rabbit-anti-KLF2 antibody | Immunoway Biotechnology | Cat# YN3112; |
| Rabbit-anti-KLF4 antibody | Proteintech | Cat# ab157157; |
| Rabbit-anti-HA-tag antibody | Proteintech | Cat# 51064-2-AP; |
| Mouse-anti-DDDDK-tag antibody | MBL International | Cat# M185-3L; |
| Alexa Fluor 680-AffiniPure Goat Anti-Mouse IgG (H+L) | Jackson ImmunoResearch Labs | Cat# 115-625-146; |
| Alexa Fluor 790-AffiniPure Goat Anti-Mouse IgG (H+L) | Jackson ImmunoResearch Labs | Cat# 115-655-146; |
| Alexa Fluor 790-AffiniPure Goat Anti-Rabbit IgG (H+L) | Jackson ImmunoResearch Labs | Cat# 111-655-144; |
| Normal Rabbit IgG Antibody | Cell Signaling Technology | Cat# 2729； |
| Human 20S Proteasome protein | R&D systems | Cat# E-360-050; |
| REGγ | This paper | N/A |
| **Bacterial and virus strains** | | |
| Adeno-associated virus: REGγ shRNA and shN | This paper | N/A |
| **Experimental models: Cell lines** | | |
| HEK293T cell | ATCC | Cat# CRL-3216; |
| WT primary adipocytes from iWAT | This paper | N/A |
| *REGγ* KO primary adipocytes from iWAT | This paper | N/A |
| *REGγ*^fl/fl^ primary adipocytes from iWAT | This paper | N/A |
| *REGγ* AKO primary adipocytes from iWAT | This paper | N/A |
| **Experimental models: Organisms/strains** | | |
| Mouse: C57BL/6J | Jackson Laboratories | Cat# JAX 000664; |
| Mouse: *REGγ* KO | a kind gift from Dr. John J. Monaco | N/A |
| Mouse: *REGγ*^fl/fl^ | This paper | N/A |
| Mouse: *REGγ* AKO | This paper | N/A |
| Mouse: LSL-*REGγ*^fl/fl^ | This paper | N/A |
| Mouse: *REGγ* AOE | This paper | N/A |
| **Chemicals, peptides, and recombinant proteins** | | |
| Dulbecco's Modified Eagle's Medium (DMEM) | Thermo Fisher | Cat# C11995500BT |
| Fetal Bovine Serum（FBS） | Gibco | Cat# 10270–106 |
| Penicillin-Streptomycin | Gibco | Cat# 15070063 |
| Lipofectamine3000 Transfection Reagent | Thermo Fisher | Cat# L3000001 |
| Insulin | Sigma-Aldrich | Cat# 11061-68-0 |
| 3-Isobutyl-1-methylxanthine（IBMX) | MedChemExpress | Cat# HY-12318 |
| T3 (3,3’,5-Triiodo-L-thyronine) | MedChemExpress | Cat# HY-A0070A |
| Rosiglitazone | MedChemExpress | Cat# HY-17386 |
| Dexamethasone phosphate disodium | MedChemExpress | Cat# HY-B1829A |
| Type II collagenase | Sigma-Aldrich | Cat# C6885 |
| RNAiso Plus | Takara | Cat# 9108 |
| HiScript II Q RT SuperMix for qPCR | Vazyme | Cat# R222-01 |
| ChamQ Universal SYBR qPCR Master Mix | Vazyme | Cat# Q711-02 |
| Cycloheximide （Chx） | MedChemExpress | Cat# HY-12320 |
| ML385 | MedChemExpress | Cat# HY-100523 |
| Pierce^TM^ protease and phosphatase inhibitor Mini Tablets， EDTA-Free | Thermo Fisher | Cat# A32961 |
| **Critical commercial assays** | | |
| Rabbit IgG-immunohistochemical SABC kit | Boster | Cat# SA1022 |
| Highly sensitive ABC immunohistochemical kit（anti-mouse IgG） | NeoBioscience | Cat# VAM100 |
| TNT® Quick Coupled Transcription/Translation System | Promega | Cat# L1170 |
| Daul-Luciferase reporter assay system | Promega | Cat# E1910 |
| **Oligonucleotides** | | |
| Primers for qRT-PCR, sequence provided in Supplementary Table 1 | This paper | N/A |
| Primers for Genotype identification, sequence provided in Supplementary Table 1 | This paper | N/A |
| **Software and algorithms** | | |
| ImageJ (version 1.8.0) | National Institutes of Health (NIH) | https://imagej.net/Welcome |
| GraphPad Prism 8.0 | GraphPad Software Inc. | https://www.graphpad.com/ |
| Adobe Illustrator | Adobe Illustrator (Ai) | https://www.adobe.com/products/illustrator.html |
| Image Studio Software | LI-COR Biotechnology | <https://www.licor.com/bio/empiria-studio/> |
| AccuFat-1050 | Mag-med | N/A |
| Metabolic Chamber-Comprehensive Lab Animal Monitoring System (CLAMS) | Columbus Instruments | N/A |
| Roche LightCycler480 | Roche | N/A |
| Temperature-controlled incubator | NK system | N/A |

**Supplementary Table 3**

| **siRNA sequence for genes** | |
| --- | --- |
| Gene | siRNA sequence(5'-3'） |
| *m-REGγ-1#* | GGAAGTGAAGCTCAAGGTTGA |
| *m-REGγ-2#* | AGAGAGCGGATCACAAGTGAG |
| *m-Nrf2-1#* | CCCGAATTACAGTGTCTTAAT |
| *m-Nrf2-2#* | CAGGACTACAGTCCCAGCA |
| *m-Acadm-1#* | CCTGGGAACGTTCGATGCTTGTTTA |
| *m-Acadm-2#* | GCCTGTGATTCTTGCTGGAAATGAT |
| *m-Klf15-1#* | CCCAGCTTCTAGTCAACATTT |
| *m-Klf15-2#* | GCAGCATTTTGGATTTCCTTT |
| *m-NF-kB-1#* | AGGCCATATAGCCTTACTATC |
| *m-NF-kB-2#* | CTGTCCTCTCACATCCGATTT |
| m-AP-1-1# | GGCATGTGCTGTGATCATTTA |
| m-AP-1-2# | ACGCAGCAGTTGCAAACGTTT |
